# Duets recorded in the wild reveal that interindividually coordinated motor control enables cooperative behavior

**DOI:** 10.1038/s41467-019-10593-3

**Published:** 2019-06-12

**Authors:** Susanne Hoffmann, Lisa Trost, Cornelia Voigt, Stefan Leitner, Alena Lemazina, Hannes Sagunsky, Markus Abels, Sandra Kollmansperger, Andries Ter Maat, Manfred Gahr

**Affiliations:** 1 Department of Behavioural Neurobiology, Max Planck Institute for Ornithology, Eberhard-Gwinner-Strasse 6a, 82319 Seewiesen, Germany; 2 Department of Zoology and Entomology, University of Pretoria, Private Bag X20, Hatfield, 0028 South Africa; 3 Graduate School of Systemic Neurosciences, Ludwig-Maximilians-Universität München, Großhaderner Strasse 2, 82152 Planegg-Martinsried, Germany; 4 Faculty of Biology, Ludwig-Maximilians-Universität München, Großhaderner Strasse 2, 82152 Planegg-Martinsried, Germany

**Keywords:** Motor control, Birdsong, Social behaviour, Cooperation, Animal behaviour

## Abstract

Many organisms coordinate rhythmic motor actions with those of a partner to generate cooperative social behavior such as duet singing. The neural mechanisms that enable rhythmic interindividual coordination of motor actions are unknown. Here we investigate the neural basis of vocal duetting behavior by using an approach that enables simultaneous recordings of individual vocalizations and multiunit vocal premotor activity in songbird pairs ranging freely in their natural habitat. We find that in the duet-initiating bird, the onset of the partner’s contribution to the duet triggers a change in rhythm in the periodic neural discharges that are exclusively locked to the initiating bird’s own vocalizations. The resulting interindividually synchronized neural activity pattern elicits vocalizations that perfectly alternate between partners in the ongoing song. We suggest that rhythmic cooperative behavior requires exact interindividual coordination of premotor neural activity, which might be achieved by integration of sensory information originating from the interacting partner.

## Abstract (web summary)

Recording neural activity during coordinated behaviors in controlled environments limits opportunities for understanding natural interactions. Here, the authors record from freely moving duetting birds in their natural habitats to reveal the neural mechanisms of interindividual motor coordination.

## Introduction

Avian duetting is a rare phenomenon, which is mainly found among bird species of the southern hemisphere. Duet songs are generally defined as overlapping bouts of sounds produced by both members of a pair^1^. Avian duets show a high diversity in complexity and in precision of coordination between the partners’ vocal emissions. While in some bird species vocalizations from both duet partners temporally overlap to a variable degree^2–4^, other birds produce vocal duets in which the partners’ contributions alternate almost perfectly^5–7^. Growing evidence suggests that alternating vocalizations in avian duets are a direct result of the partners’ effort to avoid signal overlap^2,8^. Although the degree of temporal coordination of vocal activity seems to be an important indication of the function of duetting, temporal properties of avian duets have previously rarely been quantified in detail.

A well-studied duetting songbird species is the white-browed sparrow-weaver, *Plocepasser mahali*, which is native to eastern and southern Africa. This cooperatively breeding species lives in mixed-sex groups consisting of a dominant breeding pair and up to eight subordinates^9,10^. All group members defend a year-round territory by producing highly coordinated duet and chorus songs throughout the day^11^. Duet songs of male and female *P*. *mahali* consist of introductory syllables sung by either of the partners and duet syllables emitted by both birds in a rapid but precisely timed fashion^12^. The temporal dynamics of male and female vocal productions during duetting are, however, unknown.

Another large gap in knowledge exists regarding the neural mechanisms mediating the precise interindividual coordination of vocalizations during duetting^13^. In birds, the production of song is controlled by an interconnected network of sensory and premotor brain nuclei, the song-control system^14^. Nucleus HVC represents a major relay station within this distributed, recurrent network^15^, receives auditory information^16,17^, and generates temporally patterned premotor commands for vocal production^18–20^. During singing, individual HVC neurons are active at specific time points in the song without any temporal relation to certain parameters of single song elements, such as syllable onset^21^. In anesthetized songbirds, HVC neurons show selective responses to auditory presentations of the bird’s own vocalizations^22–24^. This pattern does not seem to apply to birds that are able to produce vocal duets. HVC neurons in anesthetized plain-tailed wrens (*Pheugopedius euophrys*), a songbird species known to sing well-coordinated duets, are not only responsive to the bird’s own part of the duet but also to the partner’s vocalizations. Most importantly, neurons in the wren’s HVC respond strongest to presentations of the complete duet sequence^25^. Fortune et al.^25^ suggest that the auditory information from both duet partners might be important for the precise coordination of vocalizations during duetting. This assumption can, however, only be tested by neural recordings in the HVC of actively duetting birds. In awake individuals of nonduetting songbird species, HVC activity is mainly premotor, and responses to auditory stimulation are suppressed during singing^26–28^. Only during nonsinging periods, HVC can be responsive to playbacks of the bird’s own song^28^. From this knowledge, two alternative scenarios arise that could be present in HVC during duetting: (1) As in most nonduetting songbirds, HVC in actively duetting birds only shows premotor activity correlated to the bird’s own vocal productions and is silent to auditory input, such as the partner’s contributions to the duet. (2) In contrast to nonduetting songbirds, HVC neurons in duetting birds show premotor activity when the bird itself is vocalizing and auditory activity to the partner’s vocalizations.

Investigating the neural mechanisms that underlie an animal’s natural behavior is the fundamental aim of neuroethology^29^. Neurophysiological experiments are, however, usually conducted inside laboratories with caged animals limited in their ability to behave naturally^30^. Recent advances in the development of neurophysiological research methods have started to allow neural recordings in single laboratory animals behaving freely for a limited time within a confined outdoor area^31^. Here we present extracellular neural data that have been synchronously recorded for several days from pairs of socially interacting wild animals while they ranged completely free in their natural habitat. We exploited a self-developed radio-telemetric recording technique^32–34^ to investigate the neural basis of duet singing. We show that vocalizations in pairs of wild *P*. *mahali* precisely alternate during duetting. As in nonduetting birds, the neural activity in HVC of *P*. *mahali* is exclusively premotor during singing. The auditory information generated by the duet partner, however, alters the temporal parameters of HVC activity in the duet-initiating bird and thus enables the birds to alternate their vocalizations. We therefore conclude that in *P*. *mahali*, the integration of auditory information originating from the interacting partner mediates the precise interindividual coordination of vocal motor programs, which is required to generate precisely coordinated duet songs.

## Results

### Duetting birds precisely alternate their vocalizations

**Fig. 1:**
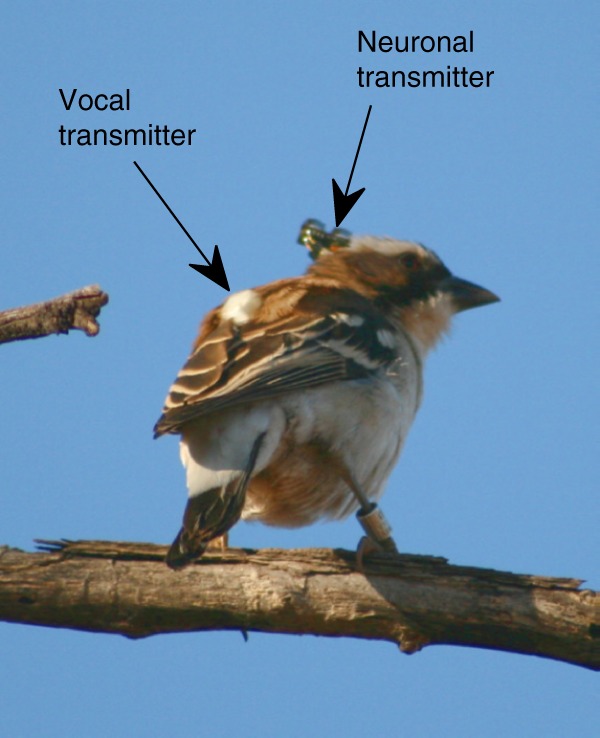
Experimental animal species. Free-living male white-browed sparrow-weaver (*P*. *mahali*) perching in a tree in the South African Kalahari. The bird carries a vocal and a neuronal (see below) radio telemetric transmitter

Instead of directional microphones that are conventionally used to monitor acoustic behavior, we fitted miniature microphone transmitters^32^ onto the backs of both partners of the *P*. *mahali* pairs (see “Methods”; Fig. 1; Supplementary Fig. 1c) and recorded the individual vocalizations of both partners during duetting in parallel. This allowed us to unambiguously assign each single vocalization to the one bird it was emitted from while preserving the precise temporal relationship between both partner’s vocalizations. In contrast to studies that used a single microphone to record vocalizations of duetting birds, our onboard microphones provided data with a much higher temporal precision since changes in distance between the birds and the microphone were nonexistent.

**Fig. 2:**
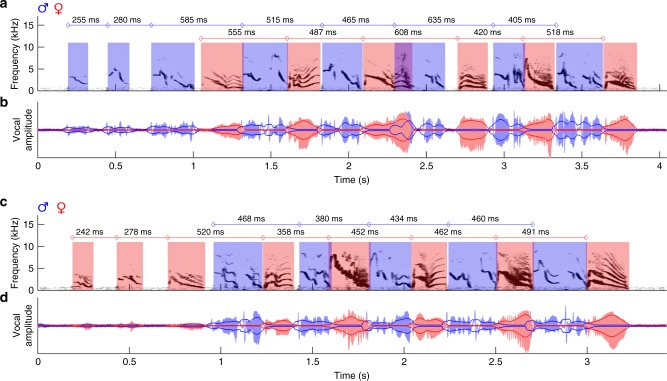
Male and female syllables alternate precisely during duetting. The spectrogram (**a**, **c**) and amplitude waveform (**b**, **d**) of the combined male and female vocal traces are shown for exemplary duet bouts initiated by the male (**a**, **b**) or by the female (**c**, **d**) of Pair #4. Male signals are coded in blue and female signals are coded in red. Solid dark blue and dark red lines outline the root-mean-square envelope (see “Methods”) of the amplitude waveforms. Onset–onset intervals of male and of female duet syllables are given by values above the spectrograms

**Fig. 3:**
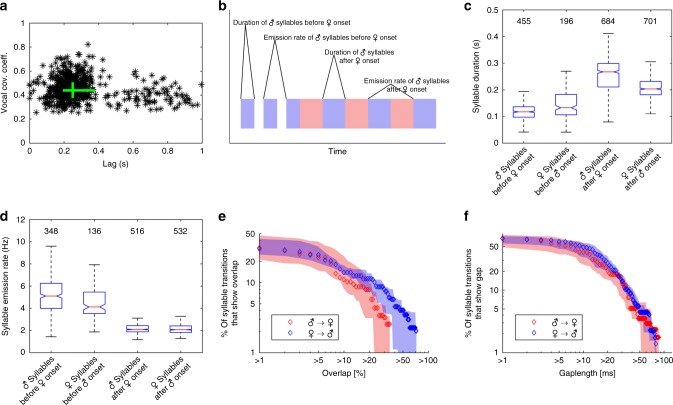
Quantification of temporal properties of vocal activity during duetting. **a** Time lags of maximum cross-covariance between male and female vocal signals (root-mean-square envelopes) during 554 duet bouts produced by 8 bird pairs were clustered at approximately 250 ms. The green lines indicate interquartile ranges of lags and cross-covariance coefficients, and their intersection is at the medians of the distributions. **b** Cartoon of a male-initiated duet bout defining the temporal duet properties that are displayed in **c**, **d**. Blue and red rectangles represent male and female duet syllables, respectively. **c** The duration of syllables sung by a single bird before the second bird’s song onset was significantly shorter than the duration of syllables sung in alternation after the second bird’s song onset. Furthermore, male duet syllables were significantly longer in duration than female duet syllables. In the boxplot, the horizontal red line indicates the median, and the bottom and top edges of the box indicate the 25th and 75th percentiles, respectively. The black whiskers extend to the most extreme data points not considered outliers (outliers are not shown). The extremes of the two notches of the box correspond to *y* − 1.57(*z* − *x*)/√*n* and *y* + 1.57(*z* − *x*)/√*n*, where *y* is the median, *x* and *z* are the 25th and 75th percentiles, respectively, and *n* is the number of observations. Medians are significantly different at the 5% significance level if the boxes’ notches do not overlap. **d** The emission rate of syllables sung by a single bird before the second bird’s song onset was significantly higher than the emission rate of syllables sung in alternation after the second bird’s song onset. Labeling of boxplots as in **c**. The median (seven pairs) percentage of duet syllable transitions that showed an overlap (**e**) or a gap (**f**) larger than the value on the *x* axis is marked by blue and red diamonds for female to male and male to female transitions, respectively. Shaded areas indicate the distributions’ interquartile ranges

In total, we analyzed 647 duet bouts produced by eight *P*. *mahali* pairs. In accordance with previous work^12^, we found that duet bouts of *P*. *mahali* generally consisted of syllables sung by a single bird to initiate duetting, followed by male and female syllables that built the actual duet (see Fig. 2 for examples). As quantitative data were highly consistent among investigated pairs, in the following, we present quantitative data for the whole population of investigated birds but provide data from individual pairs in the Supplementary Information. The timing of vocalizations during duetting was strongly correlated between partners with maximal covariance of the root-mean-square (RMS) envelopes of male and female vocal signals at a median time shift of 250 ms (interquartile range: 193–377 ms; Fig. 3a, Supplementary Fig. 3). This indicates that the birds accurately synchronized their vocal output with a phase shift of approximately 250 ms, which conforms to the average duration of male and female duet syllables (see below, Fig. 3c, Supplementary Fig. 4) and is therefore a sign of the precise alternation (Supplementary Fig. 2) of male and female vocalizations.

### Temporal fine structure of sparrow-weaver duet bouts

In 170 randomly chosen duet bouts produced by seven *P*. *mahali* pairs, we determined the onset and offset of each syllable to analyze the temporal fine structure of duet songs (see “Methods”, Fig. 3b). Duet bouts were initiated by either sex, but male-initiated bouts (114 of 170) were more common. The partner joined the song of the duet-initiating bird after, on average, four duet-initiating syllables (range: two to nine). Duet bouts were terminated by male (in 86 of 170 bouts) or female birds (in 84 of 170 bouts).

While male duet syllables (646 of 684) generally consisted of a doublet of male song elements separated by a short (median: 31.6 ms) interval of silence, female duet syllables always consisted of a single female song element. During the initiating part of the duet prior to the second bird’s song onset, male syllables were of significantly (one-sided Mann–Whitney *U* test, *p* < 0.005) shorter duration (median: 118 ms, interquartile range: 98–136 ms) than female syllables (median: 133 ms, interquartile range: 106–183 ms; Fig. 3c; Supplementary Fig. 4). In contrast, during the alternating part of the duet after the second bird’s song onset, male syllables were of significantly (one-sided Mann–Whitney *U* test, *p* < 0.005) longer duration (median: 267 ms, interquartile range: 211–299 ms) than female syllables (median: 204 ms, interquartile range: 182–232 ms; Fig. 3c; Supplementary Fig. 4).

In accordance with the short duration of male duet-initiating syllables, these syllables were produced at significantly (one-sided Mann–Whitney *U* test, *p* < 0.005) higher vocal emission rates (median: 5.1 Hz, interquartile range: 3.99–6.24 Hz) than female duet-initiating syllables (median: 4.12 Hz, interquartile range: 3.52–5.46 Hz; Fig. 3d, Supplementary Fig. 5). Counterintuitively, even though male duet syllables were of longer duration than female duet syllables, both sexes produced duet syllables at an equal rate of ~2 Hz (male median: 2.09 Hz and interquartile range: 1.87–2.43 Hz; female median: 2.06 Hz and interquartile range: 1.82–2.41 Hz; Fig. 3d, Supplementary Fig. 5). Producing shorter syllables than males but at the same emission rate as males likely gives female birds the possibility to be slightly more variable in timing their vocalizations to fit them into the gaps between the male syllables. Support for this hypothesis was provided by the fact that the variance in female duet syllable emission rates was significantly larger (one-sided Ansari–Bradley test, *p* < 0.05) than the variance in male duet syllable emission rates. Additional evidence for the lack of flexibility in vocal timing in males was provided by the male vs. female difference in the frequency of extensive syllable overlaps. Duetting birds sometimes temporally mismatched vocalizations, which resulted in a partial overlap between consecutive male and female duet syllables (see Fig. 2a for an example). Syllable overlaps that were produced by male birds were significantly (two-sided Mann–Whitney *U* test, *p* < 0.05) larger (median: 11.4% and interquartile range: 5.6–27%) than syllable overlaps produced by female birds (median: 9.1% and interquartile range: 5.1–18.2%). In more detail, small overlaps of up to 7% of the duration of the preceding duet syllable were rather common in both sexes (present in 20–30% of all syllable transitions). Overlaps >20% of the duration of the preceding duet syllable were rare, but if present, they tended to be more often produced by male (in 3–11% of all female-to-male transitions) than by female birds (in 2–6% of all male-to-female transitions; Fig. 3e, Supplementary Fig. 6). Interestingly, female but not male duet syllables that were overlapped by the partner’s following duet syllable were of significantly (two-sided Mann–Whitney *U* test, *p* < 0.005) longer duration (median: 214 ms and interquartile range: 192–260 ms) than duet syllables that were not overlapped (median: 199 ms and interquartile range: 180–223 ms). In contrast to overlaps, there was no difference in the frequency of gaps produced by male and female birds during duetting. In both the male to female and the female to male syllable transitions, short gaps between syllables were common, and long gaps were rare (Fig. 3f, Supplementary Fig. 7). Both large overlaps and long gaps occurred throughout the duet bout and were not restricted to the end of the bout, indicating that, even though the temporal pattern of the duet bout was altered by the mismatch, neither overlaps nor gaps resulted in a termination of the duet bout. Interestingly, only male birds compensated for overlaps by advancing the next duet syllable after being overlapped by a female. The median onset–onset interval of male but not of female duet syllables was significantly smaller (two-sided Mann–Whitney *U* test, *p* < 0.005) after an overlap compared to the intervals between non-overlapped duet syllables.

### Mechanisms for vocal coordination between duetting birds

Above, we demonstrated that during duetting both partners of *P*. *mahali* pairs precisely coordinated their vocal emissions. The coordination of motor actions between individuals requires an external common event that defines the onset of coordinated behavior. In *P*. *mahali*, syllables sung solo by the duet-initiating bird before the second bird’s song onset were produced at a median rate of 5.1 and 4.1 Hz by male and female birds, respectively (Fig. 3d; Supplementary Fig. 5). After the second bird’s song onset, however, male and female duet syllables were produced at a significantly (two-sided Mann–Whitney *U* test, *p* < 0.005) lower rate (median emission rate: 2.1 Hz for males and females; Fig. 3d; Supplementary Fig. 5). This reduction by approximately half of the initial vocalization rate maintained the overall rate of male and female syllables in the alternating part of the duet bout constant at ~4 Hz. The sudden change in vocalization rate of the duet-initiating bird immediately after the partner joined the duet suggests that the auditory input generated by the partner’s first duet syllable triggered the change in song rhythm in the duet-initiating bird. The bird that joined the duet immediately vocalized at a low rate of ~2 Hz. We therefore hypothesize that the song onset of the bird that joins the duet represents the common cue that defines the onset of vocal coordination in both birds.

Once initiated, the vocal coordination could either be maintained passively by each bird of a pair singing its own part of the duet with a temporally fixed pattern and without paying attention to the partner’s vocalizations or actively by both birds fine-tuning the timing of their vocalizations to those of the partner. The first mechanism would be highly prone to external disturbances; for example, slight variations in the song rhythm of one bird would result in a breakdown of coordination. The latter mechanism would, however, allow the birds to compensate for small temporal irregularities such as syllable overlaps. We, therefore, suggest that *P*. *mahali* could actively maintain vocal coordination in the ongoing duet by one of the following three mechanisms: a bird locks the onset of its own duet syllable to (A) the onset of the partner’s preceding duet syllable, (B) the offset of the partner’s preceding duet syllable, or (C) the point in time when it has recognized the type of syllable the partner was currently vocalizing. In *P*. *mahali* of both sexes, we found the variance in partner-onset to own-onset latencies (male median latency: 219.2 ms and interquartile range: 187.9–258.5 ms; female median latency: 270.0 ms and interquartile range: 220.1–313.2 ms) to be significantly larger (one-sided Conover’s Squared Ranks Test, *p* < 0.005) than partner-offset to own-onset latencies (male median latency: 15.6 ms and interquartile range: −11.3 to 40.4 ms; female median latency: 10.4 ms and interquartile range: −15.7 to 34.2 ms). Locking one’s own syllable onset to the onset of the partner’s preceding syllable should, however, result in onset–onset latencies that vary only minimally. Therefore, we exclude hypothesis A. Although the less variable offset-onset latencies between male and female *P*. *mahali* duet syllables would support hypothesis B, the fact that the birds sometimes produced syllable overlaps (see above) led us to exclude this hypothesis too. To lock its own syllable onset to the point in time when a bird has recognized the partner’s preceding syllable type (i.e., hypothesis C) requires each bird to have knowledge about the duration of each syllable type in the partner’s repertoire since latencies would depend on the duration of the partner’s preceding syllable. Syllable overlaps would be possible in cases when the duration of the partner’s preceding syllable exceeded the anticipated duration. After excluding two of the three proposed mechanisms, we suggest that to maintain vocal coordination during duetting, *P*. *mahali* adjust the time of syllable onset to the “expected” offset of the partner’s preceding syllable (i.e., hypothesis C). One way to prove this hypothesis would be to perform syllable-type-specific analyses of *P*. *mahali* duets. Focusing on specific male–female syllable transitions eliminates variation in syllable duration and spectrotemporal composition and would therefore likely allow to determine the time point in the partner’s syllable to which a bird locks its own syllable onset. Owing to the large vocal repertoire of *P*. *mahali*, this type of analysis would, however, require a dataset comprised of several hundreds of duet songs for each pair of birds and is therefore subject of future research.

### Flexibility in vocal timing during duetting is limited

To test the degree of flexibility of vocal coordination in duetting *P*. *mahali*, we conducted playback experiments, which included presentations of manipulated duet bouts (see “Methods”). Since our dataset was too small to enable a quantitative analysis, we present here only exemplary data. In response to the playback, the birds were highly agitated and immediately started to duet with each other (Supplementary Movie 2). Sometimes, a single bird or both duet partners tried to duet with the playback. When we played back an unaltered version of the duet consisting of alternating male and female duet syllables with the normal rhythm, birds were able to follow the rhythm and produced their sex-specific syllables exactly at the same time as they were produced by the playback (Supplementary Fig. 8a). If the stimulus, however, consisted of only the male or female duet syllables with a two-fold increase or with a bisection of the length of intervals between sex-specific duet syllables (i.e., twice or half of the normal vocalization rate), the birds were no longer able to synchronize their vocal productions with the playback (Supplementary Fig. 8b, c). Although during duetting with a partner small irregularities in song rhythm could be compensated for (see above), the birds were not able to adjust their syllable emission rates to unnaturally large deviations from this rhythm while trying to duet with the playback. To determine the value of maximal deviation from the normal rhythm the birds would still be able to follow, a finer gradation of temporal manipulations of intervals between sex-specific duet syllables would be necessary.

It is generally assumed that rhythmic motor patterns are generated by the activity of central pattern generators (CPGs), which produce a rhythmically timed pattern of premotor neural activity^35,36^ that can be regulated by sensory feedback^37,38^. Nucleus HVC has repeatedly been suggested to be a part of the CPG network that controls the timing of vocalizations during birdsong^20,21,39^. Although the production of duet syllables in male and female *P*. *mahali* followed a common rhythm, duet syllable emission rates were not completely stable but varied by approximately 50% of the median and could be adjusted to small irregularities produced by the partner. Thus we suggest that the rhythmical production of vocalizations during duet singing in *P*. *mahali* is controlled by a CPG network that can be naturally modulated by the social auditory input but only to a certain degree. This assumption has been supported by studies in nonduetting songbirds showing that perturbations of the CPG network controlling vocal production by brief electric stimulation of HVC^40^ or by the presentation of altered auditory feedback^41^ during singing results in altered song patterns.

### HVC activity is locked to the bird’s own duet syllables

**Fig. 4:**
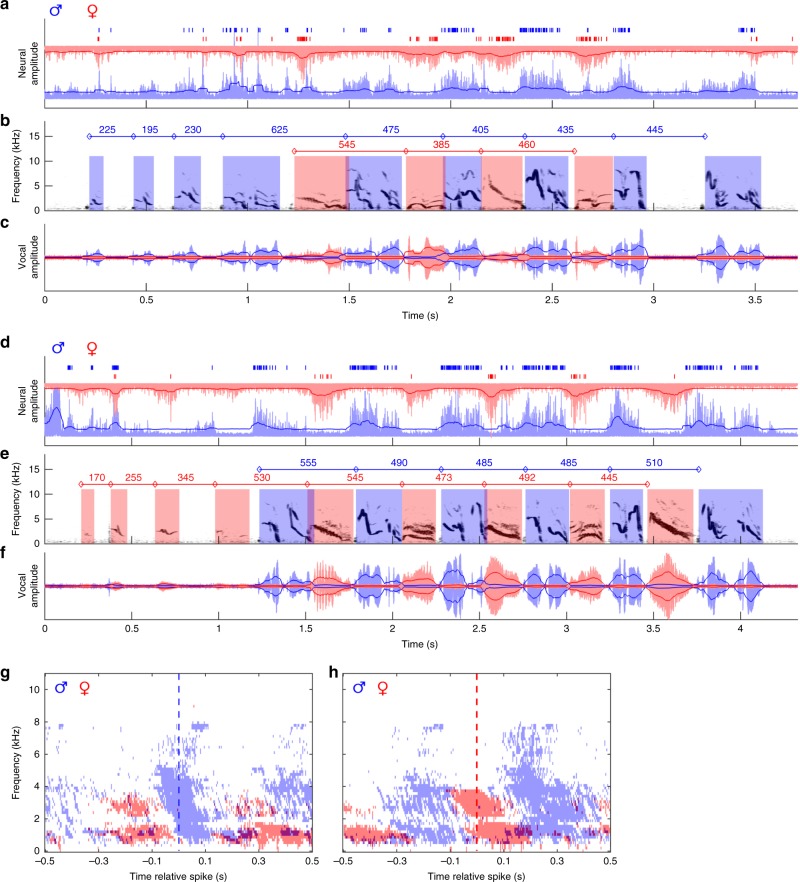
Vocalizations are locked to bursts of premotor HVC activity in the singing bird. Throughout, male signals are coded in blue and female signals are coded in red. The filtered and rectified male and female neural traces (**a**, **d**) and the spectrogram (**b**, **e**) and amplitude waveform (**c**, **f**) of the combined male and female vocal traces are shown for exemplary duet bouts initiated by the male (**a**) or by the female (**d**) of Pair #5. Solid dark blue and dark red lines outline the root-mean-square envelope (see “Methods”) of neural and vocal signals. Times of spike occurrence are indicated by short vertical lines above the neural traces. Onset–onset intervals of male and of female duet syllables are provided by values above the spectrograms. Please note the precise alternation of neural bursts between interacting males and females. Significant (*p* < 0.01, *t* test) activity in the averaged spectrogram (see “Methods”) of male and female vocal signals within a window of 500 ms before and after the time of occurrence of 3000 male (**g**) and 3000 female (**h**) spikes that occurred during 46 duet bouts of Pair #5 is shown by clusters of blue and red time–frequency pixels. The dashed blue and red lines mark the time of occurrence of male and female spikes, respectively, used for generation of the averaged spectrogram

Uncovering the high degree of precision with which vocal timing was coordinated during duetting in pairs of *P*. *mahali* raises the question of the neural substrates that enable the precise interindividual coordination of vocal productions in these birds. To find an answer to this question, we recorded extracellular neural activity in parallel with individual vocalizations during duetting in three wild *P*. *mahali* pairs in their natural habitat, the Kalahari savanna (see Supplementary Movies 1 and 2). In addition to the vocal transmitter, we equipped both birds of a pair with a radio transmitter that was connected to an electrode implanted in the bird’s HVC (see “Methods”; Fig. 1; Supplementary Fig. 1a–c). This yielded perfectly synchronized recordings of the vocal and neural activity in both individuals of the pair, which cannot be obtained when using conventional bioacoustic and neurophysiological recording techniques^32^. Most evident in the neural signal of each bird was the observation that, during duetting, multiunit activity was exclusively locked to the singing bird’s own vocalizations. During the partner’s duet syllables, activity in HVC was not increased (Fig. 4; Supplementary Fig. 9). The majority of spikes in all six birds occurred during the first half of a bird’s own vocal emissions (Supplementary Fig. 10), which indicates that the neural activity we recorded during duetting was mainly premotor and not auditory.

Although the partner’s vocalizations did not result in auditory-evoked activity in HVC neurons, it might have been possible that this auditory input had modulating effects on premotor activity. We analyzed the spike rate during the production of duet syllables that were overlapped by the partner’s duet syllable and found that HVC activity was not affected by overlaps. In both the male and female HVC, the median spike rate during overlapped syllables (male median: 23.5 and interquartile range: 8–36 spikes per syllable; female median: 12.5 and interquartile range: 5–25 spikes per syllable) did not differ from the median spike rate during nonoverlapped syllables (male median: 23 and interquartile range: 9–37.5 spikes per syllable; female median: 12.5 and interquartile range: 6–22 spikes per syllable) that preceded or succeeded the overlap. Note that the difference in the median spike rate between male and female birds was not significant (one-sided Mann–Whitney *U* test, *p* > 0.01).

### Activity in the HVC is correlated between duet partners

**Fig. 5:**
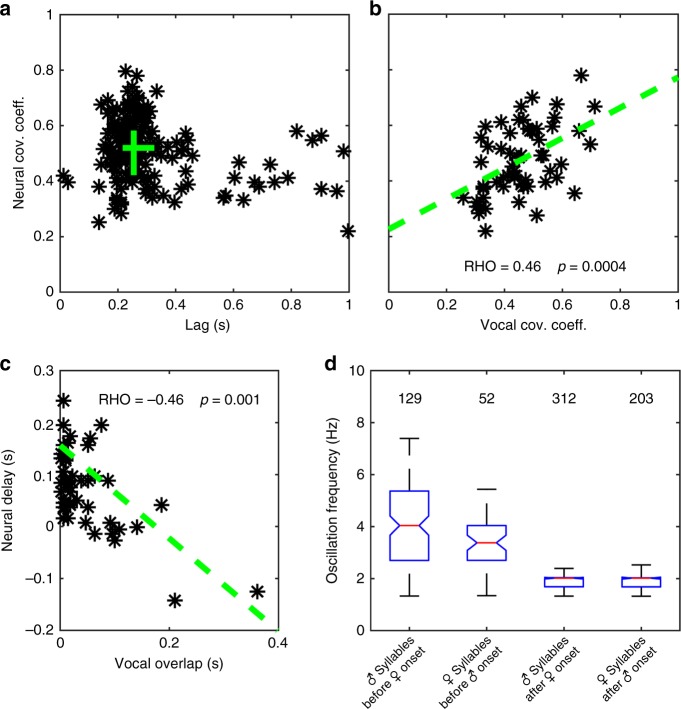
Quantification of temporal properties of HVC premotor activity during duetting. **a** Time lags of maximum cross-covariance between male and female neural signals (root-mean-square envelopes) during 179 duet bouts produced by three bird pairs were clustered at approximately 250 ms. The green lines indicate interquartile ranges of lags and cross-covariance coefficients, and their intersection is at the medians of the distributions. **b** The covariance of neural signals was strongly correlated with the covariance of vocal signals. The dashed green line represents the regression line. *n* = 56 duet bouts of three bird pairs. **c** The degree of overlap in neural activity was strongly correlated with the degree of syllable overlap. The dashed green line represents the regression line. *n* = 47 overlaps produced by two bird pairs. **d** During emission of duet-initiating syllables, oscillation frequencies of the male and female neural signals were significantly higher (*p* < 0.005, Mann–Whitney *U* test) than during emission of alternating syllables during duetting. Labeling of boxplots as in Fig. 3c

As shown above for vocal signals, male and female neural signals were strongly correlated during the alternating part of duet bouts. The covariance of the RMS envelopes (see “Methods”) of neural signals was maximal at a median time shift of 255 ms (interquartile range: 217–326 ms, Fig. 5a, Supplementary Fig. 11). Most importantly, during the alternating part of duet bouts, the degree of interindividual synchronization of neural activity was positively correlated (Spearman’s Rho: 0.46, permutation test for large-sample approximations, *p* < 0.001) with the degree of interindividual synchronization of vocal activity (Fig. 5b, Supplementary Fig. 12). This may indicate that a precise synchronization of premotor neural activity between partners is required for an exactly timed duet performance. A similar relationship between neural activity and vocal behavior was observed during syllable overlaps. The degree of syllable overlap was significantly correlated (Spearman’s Rho: −0.46, permutation test for large-sample approximations, *p* < 0.005; Fig. 5c, Supplementary Fig. 13) with the delay between the last spikes that occurred during the overlapped syllable and the first spikes that occurred during the overlapping syllable.

### The partner’s song onset affects the neural burst rate

Similar to the temporal pattern of the vocal behavior in *P*. *mahali*, we found a significant (two-sided Mann–Whitney *U* test, *p* < 0.005) decrease in oscillation frequency of HVC activity in the duet-initiating bird right after the partner had joined the duet. During the initiating part of the duet, bursts of multiunit activity in the neural signal of male and female duet-initiating birds occurred at a median rate of 4.0 and 3.4 Hz, respectively, whereas the median burst rate dropped to 2.0 Hz in both sexes immediately after the partner started contributing to the duet (Fig. 5d; Supplementary Fig. 14). This suggests that the auditory input generated by the partner’s vocalizations influences the timing of vocal premotor activity in the singing bird.

## Discussion

In contrast to conventional neurophysiological methods, our radio telemetric approach allows us to investigate the neural basis of an animal’s behavior under completely natural conditions. To date, neurophysiological studies have been conducted with laboratory animals that spend their lives in cages. The behavior of caged animals, however, very likely differs from that of free-living conspecifics as the latter regularly face situations due to weather conditions, hunger, or predation, which would never be experienced by laboratory animals and which probably have a very large impact on an animal’s natural behavior^30^. In addition, especially for animals such as *P*. *mahali* that live in groups with a sophisticated social structure, certain aspects of their behavioral ecology would be impossible to set up in a laboratory environment. Our technique now enables access to the neural activity of several group members in parallel, while each individual can behave naturally. This opens up a plenitude of possibilities to researchers who aim to study the neural substrates of complex social behaviors (e.g., collective movement or cooperative foraging) that can only be performed within a natural context.

Here we recorded individual vocalizations and premotor vocal activity in parallel from pairs of duetting birds to investigate how the precise interindividual coordination of motor activity during duet singing is neuronally controlled. Previous dual neuroimaging studies, performed in human subjects while they were engaged in rhythmic social interactions, showed that synchronized motor actions between subjects were accompanied by an overall synchronization of brain activity^42,43^. Our present study demonstrates that rhythmic social interactions that require temporal coordination are not just associated with a coherent oscillation of activity in large areas of both interacting partners’ brains^43^ but with an interindividually synchronized activation of small groups of neurons within the same brain nucleus in both partners. Our results strongly suggest a scenario in which the brains of two birds constitute a network that acts as a distributed circuit to organize the temporal pattern of vocal duets. We expect this to apply also to other rhythmic cooperative behaviors but experimental support provided by further dual-electrophysiological experiments in freely behaving animal models is required.

Our results show that, as in most nonduetting songbirds, during singing HVC activity in duetting birds is locked to each individual’s own vocalizations. This strongly contrasts with what one would expect from the findings of a previous study in plain-tailed wrens. HVC neurons in anesthetized wrens differ in their activity pattern from that of most nonduetting songbirds by showing auditory-evoked activity not only to playbacks of the bird’s own vocalizations but also to presentations of the partners song and even further increasing their activity when stimulated with a combination of both the male and female parts of this species’ duet song^25^. HVC neurons in awake and actively duetting *P*. *mahali* were, however, responsive to neither the partner’s vocalizations nor to playback of prerecorded duet bouts. It has been repeatedly shown that activity in the song-control system depends on the bird’s behavioral state^44–46^. Under anesthesia and during sleep, HVC neurons usually show strong, consistent, and highly selective auditory responses to presentations of the bird’s own vocalizations. In awake birds, however, if present, the strength of auditory responses in HVC is highly variable, and neurons are unselective for the bird’s own song. McCasland and Konishi^47^ reported for three different songbird species that auditory responses in HVC are completely suppressed by vocal motor activity during singing. More recently, Hamaguchi et al.^48^ demonstrated that, while auditory input failed to alter intracellular activity of HVC neurons during singing, the same cells responded to auditory stimulation when the bird ceased to vocalize. This may indicate that, also in the awake condition, HVC processes auditory information, but during singing, the output of this processing is subthreshold. In duetting *P*. *mahali*, we observed that auditory information generated by the partner’s syllables never elicited an auditory response but could trigger a change in vocalization rate in the duet-initiating bird. A similar observation has been made in duetting plain wrens (*Cantorchilus modestus zeledoni*), suggesting that vocal production in individuals who sing duets is controlled by autogenous and heterogeneous auditory feedback rather than by intrinsic fixed action patterns^49^. Further support for this hypothesis was provided by our finding that, during duetting, male *P*. *mahali* were able to compensate for syllable mismatches. This indicated that each bird listened to its own and the partner’s vocalizations to fine-tune the timing of vocalizations. We therefore suggest that, during duetting, the auditory information from the partner’s syllables is reaching HVC and is most likely used for the interindividual coordination of vocalizations in *P*. *mahali*. An alternative but less likely hypothesis is that, during duetting, HVC does not receive the “raw” auditory information generated by the partner but rather a premotor signal that is the result of auditory processing in downstream brain areas. Such a scenario has been demonstrated to exist in nonduetting songbirds to coordinate the premotor signals for song generated in the song-control system of both hemispheres in a way that the vocal organ is able to produce proper sounds^50^.

In most bird species that produce vocal duets, the females are assumed to lead during duetting^2,51–53^ (see ref. ^4^ for a contrary example). The leading role has been attributed to the bird of a pair that most frequently initiated duets^51–53^, produced overlaps most often^2^, determined the song rhythm^4^, i.e., showed less behavioral adaptation, or produced vocalizations that elicited the stronger neural responses in the HVC^25^. Our study, however, provides support for the assumption that male birds lead during duetting: (1) Duets were most often initiated by male *P*. *mahali*. (2) Vocal emission rates during duetting were less flexible in males than in females, which may indicate that the male was determining the song rhythm. (3) Male *P*. *mahali* produced duet syllable overlaps more often than females. Although it is well established that the emergence of leader and follower roles enhances the quality of group performance^54^, the factors that determine which partner adopts which role during joint actions are still not entirely clear. It has been suggested that the partner who maintains the rhythm becomes the leader and the partner who maintains the synchrony of the joint behavior becomes the follower, as maintaining synchrony requires greater adaptation^55^. Recently, a neural correlate of leader–follower distinctions has been described. Using dual-electroencephalogram measurements in two human participants who performed a synchronized tapping task, Konvalinka et al.^56^ showed that leaders but not followers exhibited a reduction in alpha and low-beta oscillations over motor and frontal areas, which may have reflected the increase in prospective planning and control required for maintaining the rhythm of joint actions^57^. Additional studies such as ours, which measure brain activity synchronously in pairs or groups of individuals during natural social interactions, are, however, essential to further the understanding of the neural mechanisms that underlie the coordination of joint actions.

## Methods

### Animals and ethics approval

We studied a population of wild *P*. *mahali* near the village of Black Rock, Northern Cape, South Africa (27°7′S, 22°50′E) during February/March 2016 and November/December 2017. All investigated birds were color-banded for individual recognition. The birds were captured shortly after dusk (0800–1000 p.m.) inside their roosting nests. The sex was determined by bill color^58,59^. The male bird of all investigated pairs was likely the dominant male of the group, whereas the social status of female birds could not be determined reliably.

All bird-capture permits were obtained from the Northern Cape Department of Nature Conservation. All experiments described here complied with the relevant ethical regulations for animal testing and research and were approved by the Animal Ethics Committee of the University of Pretoria. To ensure that the birds would not be restricted in their natural behavior while carrying the transmitters, we tested all devices on captive *P*. *mahali* in Seewiesen, Germany, under permit 55.2-1-54-2532-175-2016 issued by the government of Upper Bavaria. After the initial experiments in Germany had proven that the birds only show short-term (up to a few hours) effects (e.g., less singing and less locomotion than usual) while habituating to carrying the transmitters, and other aspects of their normal behavior (e.g., flight performance, feeding, entering and exiting their nests) were not affected by the transmitters, we went to South Africa. In 2016, we only recorded the individual vocal behavior of wild *P*. *mahali* pairs with microphone transmitters. For these experiments, we received ethics approval from the University of Pretoria (permit: EC086-15). In 2017, we combined the vocal recordings with telemetric neural recordings. After paying special attention to pain management, to bird recapture, and to the possible effects of the transmitting devices on the birds’ behavior, for this second set of experiments, the University of Pretoria granted us ethics approval (permit: EC026-17) bound to additional obligations (e.g., the presence of a state veterinarian during surgical interventions on the birds).

### Vocal recordings

To monitor individual vocal activity in free-ranging *P*. *mahali*, we equipped both birds of wild pairs with on-board radio telemetric transmitters developed at the Max-Planck-Institute for Ornithology in Seewiesen, Germany. The lightweight vocal transmitter (0.6 g), which included a miniaturized microphone (FG23329, Knowles Electronics, USA), was covered by a thin silicon casing and fixed on the back of the bird (Fig. 1; Supplementary Fig. 1c) with cotton-covered rubber-band straps around both femurs and the abdomen^32^. The transmission range of the device averaged 50 m and the battery life 15 days. Carrying these microphone transmitters has only small and short-term habituation effects on the vocal and movement activity of songbirds^32^. For signal detection, a crossed Yagi antenna (Winkler Antennenbau, Germany) was placed below the nesting tree in the center of the birds’ territory (Supplementary Fig. 1d). An antenna amplifier (TVS 14-00 axing, Goobay®, Germany) increased the antenna signal by 18 dB. The signal was split (BE 2-01 premium-line, Switzerland) and fed into up to eight communication receivers (AOR 8600, AOR Ltd., Japan), which were modified to handle 12 kHz audio bandwidth. The analog signals were digitized by an eight-channel audio A/D converter (M-Track Eight, M-Audio, USA; sampling rate: 22050 Hz) that was connected to a laptop computer (Supplementary Fig. 1e). All digitized signals were recorded in parallel as continuous audio files with a duration of 4 h using the multichannel software (16-bit, 22050 Hz; ASIO®, Germany). The recording set-up was placed in the trunk of a car ~30 m from the antenna (Supplementary Fig. 1f). For each pair, vocal recordings were continuously conducted over several consecutive days.

### Neural recordings

Birds that were chosen to carry the additional neural transmitter (Fig. 1 and Supplementary Fig. 1c) received analgesic treatment (meloxicam 5 mg ml^−1^, Metacam, Boehringer-Ingelheim, Germany; 0.2–0.5 mg kg^−1^) prior to anesthetization with isoflurane inhalation (isoflurane 1.5–1.8% in 0.5 l O_2_ min^−1^, IsofluranCP, CP-pharma, Germany). The birds were wrapped in a thin gauze blanket and kept warm by warm water bags (Supplementary Fig. 1b). The skin of the head was treated with lidocaine liquid (lidocainhydrochlorid, Minocain, BelaPharm, Germany), plucked, and disinfected with alcohol swabs (Henry Schein, Germany). The skin was opened in rostro-caudal direction along the midline. A first craniotomy was performed to localize the bifurcation of the midsagittal sinus, which served as stereotaxic reference point, and a second craniotomy and a durotomy were performed over the HVC. Stereotaxic coordinates had been ascertained beforehand in captive animals. In all birds, the recording electrode was lowered into the HVC at a lateral distance of 2600 µm from the reference point using a piezo single-axis micromanipulator (SMX series, SENSAPEX, Sweden) that was mounted on a stereotaxic frame (WPI, USA). The reference electrode was inserted between the skull and dura mater. During electrode implantation, the recorded signal was amplified using an AC differential amplifier (DAM 80, WPI, USA), filtered between 200 and 10,000 Hz by a custom-made, dual state variable filter (Free University of Amsterdam, The Netherlands), digitized by a battery-operated USB audio interface (Duo-Capture SX, Roland, Germany) and monitored online by a laptop using a continuous update of the interspike interval of Schmitt-triggered spikes. Photographs of the set-up used to implant electrodes are shown in Supplementary Fig. 1a, b.

After detecting stable auditory-driven neural activity with a good signal-to-noise ratio, the electrode was fixed (at depths between 217 and 360 µm below brain surface) by gluing the connector pins of the reference and the recording electrode to the skull using dental cement (Tetric evoflow, Ivoclar Vivadent, Liechtenstein), and a self-made radio-telemetric transmitter (weight: 1.0 g) was connected to the pins^33,34^. The transmission range of the device covered >50 m, with a battery life of >7 days. The bandwidth of the transmitter’s amplifier stage ranged between 10 and 11,000 Hz to enable recording of local field potentials as well as spiking activity. The transmitter was connected to a single parylene-coated tungsten electrode (FHC, USA; impedance: 2 MΩ) and a reference electrode (platinum wire with a diameter of 50 µm, Advent Research Materials, UK).

After the surgical intervention, the birds were allowed to recover in cloth cages until dawn. All birds recovered quickly from the treatment, and we were able to release them to their home territory a couple of hours after the surgical implantation of the electrode and mounting of the transmitters. This protocol was essential to our study since we had observed that removing birds from their group during the day could destroy the group’s social structure. By releasing the birds soon after the treatment (shortly before sunset), we could guarantee that all treated birds readily reintegrated into their group and maintained their social position. Before release at civil twilight, we administered a second analgesic injection (Metacam 0.2–0.5 mg kg^−1^, Boehringer-Ingelheim, Germany). Recording of neural and vocal activity (Supplementary Movies 1 and 2) started as soon as the birds were released. Whenever the birds were in the reception range of the antenna, the individual vocalizations and neural activity of both birds of a pair were telemetrically recorded in parallel for up to 4 days as described above. It is important to note that the position of the recording electrode was fixed in each bird. This means that we recorded for several days from one site in the HVC per bird.

Constant monitoring of the treated birds certified that they did not show any sign of discomfort or altered behavior due to the treatment. All but one female bird remained at their colony for the entire experiment. At the end of the experiments, the birds were re-trapped, and the transmitters were dismounted. Birds that carried neural transmitters were either released to their home territory (*n* = 5) or euthanized for brain sampling by an overdose of isoflurane after applying an electrolytic lesion at the recording site (*n* = 6). Only pairs of birds in which both neural transmitters provided stable signals over 2 consecutive days were euthanized since only data from those birds could be used for analysis. Electrolytic lesioning was performed by connecting both the reference and the recording electrode to the DAM 80 amplifier. Timing (6 s) and current (4.5 µA) of the lesions were adjusted according to Ter Maat et al.^34^. To verify electrode placement, dissected brains were transferred to 4% paraformaldehyde and transported to the Max-Planck-Institute for Ornithology. Here the brains were cryocut in 30 µm sections and stained with thionin. Supplementary Fig. 15 shows a sagittal brain section of a male *P*. *mahali* with a lesion in the HVC.

### Acoustic playback

During the experiments in 2017, we presented playback of prerecorded duet songs to the experimental birds, for at least 30 min a day. The playback was broadcast by a battery powered active speaker (Mobile BA, ROLAND, Germany) that was placed in close vicinity to the roosting tree of the respective pair. We used four different duet song bouts in seven different versions: complete duet, male part only, male part only with prolonged gaps between syllables, male part only with shortened gaps between syllables, female part only, female part only with prolonged gaps and female part only with shortened gaps. All song versions were normalized to 3 dB of the maximum and bilaterally faded. Supplementary Movie 2 shows a pair of *P*. *mahali* duetting with the playback. In parallel with the vocal and neural signals of the birds that were equipped with transmitters, the playback signal was rerecorded by the use of a microphone transmitter located close to the speaker.

### Sound analysis

The temporal fine structure of duet bouts for each studied pair of *P*. *mahali* was analyzed by different persons to avoid biasing the measurements. For each bird, all 4-h sound files were initially inspected audiovisually with the software Amadeus® or Audacity® to determine the time of duet bout occurrences. Based on Voigt et al.^12^, a duet bout was defined as a sequence of introductory song syllables followed by male and female duet syllables. Each duet bout was surrounded by at least 1.5 s of silence and duet bouts temporally overlapped in the vocal signals of the male and female bird of a pair. The time point of the start of the first syllable and the end of the last syllable of each duet bout was noted. To obtain general timing properties, such as syllable duration and syllable interval, the start and the end of each female syllable and each male syllable element were measured in a subset of 15 to 20 duet bouts for each pair of birds. This approach follows the method used in Thorpe et al.^60^ to define the reaction time in duetting pairs. Onsets and offsets of vocal elements were defined by a deviation of the vocal signal from baseline activity by 5%. The onset of the first syllable element and the offset of the second syllable element in the male duet syllables served as the onset and offset for male syllables, respectively.

In addition, a semiautomated method was used to quickly determine the onsets of female syllables and male syllable elements in all recorded duet songs. Sonograms from vocal signals were assembled from 512 point Fast Fourier Transforms (Intel Libraries) using custom written software (R.F. Jansen; Delphi Pascal for Windows, and Andries Ter Maat; CodeWarrior and C++ for Mac OS X). Each sonogram describes a syllable or any other suprathreshold sound. To determine the average activity pattern in the vocal signals of each bird of a pair in relation to syllable occurrence in the vocal signal of one of the birds, syllable-triggered averaged spectrograms were generated for each bird using a reverse correlation technique. For this, the onset of each syllable in each duet bout was extracted from the data set. In accordance with a method described by Jenison et al.^61^, for each syllable onset, the spectrogram (FFT length: 128, overlap: 50, Hamming window; MATLAB®, Mathworks, USA) of the vocal signal of each individual of a pair was calculated within a 500-ms segment preceding and succeeding the syllable onset. To evaluate the level of uncorrelated background activity, spectrograms were constructed from randomly drawn 1-s segments of each vocal signal at random positions within a period of 3 s before and 3 s after the syllable onset^62^ and significant deviations from the background activity were determined by a pixelwise *t* test^63^. In the final display of the syllable-triggered average, significant (*p* < 0.01) pixels in the male and female vocal signals were indicated in blue and red, respectively. Each syllable-triggered average was based on at least 250 syllables that occurred during all duet bouts analyzed for a particular pair of birds. For each syllable-triggered average, the number of randomly drawn segments was equal to the number of syllable-triggered segments.

ITo determine the correlation between vocal signals of both duet partners, we measured the peak and the time lag at the peak of the cross-covariance function (MATLAB®) between the upper RMS envelopes (MATLAB®) of the signals for each duet bout. The envelopes were determined using a sliding window of a length of 1000 samples. During duetting, the male and the female bird often sat close together, which theoretically opened up the possibility that the microphone of the vocal transmitter of one bird picks up the vocal signal of the partner. However, since the receptive part of the microphone was directed toward the body surface of the bird it was mounted on, and the transmitter’s plastic cover and the bird’s feathers further shielded the microphone from external sound sources, the partner’s vocalizations produced only negligible amplitude variations in the vocal traces, which were never detected by our sound analysis software and were averaged out in the RMS envelopes used for covariance analysis.

### Neural analysis

ITo extract spike times from the continuous neural signals of each bird, each neural trace was initially bandpass filtered (200–4000 Hz, fourth order Butterworth) using the MATLAB® filtfilt function. Supplementary Fig. 16 shows examples of raw and filtered neural traces temporally aligned to vocal activity. A threshold for discriminating spikes from background activity was determined by averaging (MATLAB®) the mean plus three standard deviations of the neural signal during each duet bout within a 4-h sound file. The filtered neural signals were then fed into the software Spike2® (CED, UK), and spikes were discriminated from background activity by setting a threshold to the value calculated as described above (see Supplementary Fig. 16 for an example). The average signal-to-noise ratios (singing vs. non-singing) of filtered neural signals ranged between 3.5 and 9.6 dB, and average spike rates during singing and non-singing periods ranged from 25.0 to 113.3 spikes s^−1^ and from 5.7 to 35.1 spikes s^−1^, respectively (see Supplementary Table 1). To examine the temporal relationship between the vocal and neural signals and to exclude the presence of artifacts, for each bird we plotted mean activity profiles of both signals aligned to syllable onset for all syllables produced within a 4-h recording period (see Supplementary Fig. 17 for examples).

To determine the averaged activity in the vocal signals of both partners in relation to spike occurrences in the neural signal of one partner, spike-triggered averaged spectrograms were generated for each bird. For each spike, the spectrogram (FFT length: 128, overlap: 50, Hamming window; MATLAB®, Mathworks, USA) of the vocal signal of each individual of a pair was calculated within a 500-ms segment preceding and succeeding the time of spike occurrence. To evaluate the level of uncorrelated background activity, spectrograms were constructed from randomly drawn 1-s segments of each vocal signal at random positions within a period of 3 s before and 3 s after spike occurrence^62^ and significant deviations from the background activity were determined by a pixelwise *t* test^63^. In the final display of the spike-triggered average, significant (*p* < 0.01) pixels in the male and female vocal signal were indicated in blue and red, respectively. Each spike-triggered average was based on at least 400 spikes, and for each spike-triggered average, the number of randomly drawn segments was equal to the number of spike-triggered segments.

To determine the correlation between neural signals of both duet partners, we measured the peak and the time lag at the peak of the cross-covariance function (xcov, MATLAB®) between the upper RMS envelopes (envelope, MATLAB®) of the signals for each duet bout (see above). To measure the oscillation frequency of multiunit bursts in the neural signal of one bird before and after the song onset of the second bird, we calculated the autocovariance function (xcov, MATLAB®) between the upper RMS envelopes (MATLAB®) of the neural signal for both parts during each duet bout and performed a spectral analysis of the function by calculating the FFT (MATLAB®) with an input length that was the next power of two from the original signal length. The single-sided amplitude spectrum was plotted for the frequency range (1–10 Hz) that we expected to include the oscillation frequency of the signal, and the frequency at the peak of the spectrum was noted.

### Reporting summary

Further information on research design is available in the Nature Research Reporting Summary linked to this article.

## Supplementary information

Supplementary Information

Reporting Summary

Description of Additional Supplementary Files

Supplementary Movie 1

Supplementary Movie 2

## Data Availability

The data that support the findings of this study are available from the corresponding author upon reasonable request. Owing to the large size of data files, they are not publicly available. A reporting summary for this article is available as a Supplementary Information file.

## Code availability

All custom computer codes used to generate results that are reported in the paper are available from the corresponding author upon reasonable request.
