# Supplementary material for: Duets recorded in the wild reveal that interindividually coordinated motor control enables cooperative behavior
Source: Nat Commun. 2019 Jun 12;10:2577. doi: 10.1038/s41467-019-10593-3 (PMC6561963; doi:10.1038/s41467-019-10593-3)
Supplement: Supplementary file 1 — Supplementary Information [file 41467_2019_10593_MOESM1_ESM.pdf]

Supplementary Information

Duets recorded in the wild reveal that interindividually coordinated motor control enables cooperative behavior

Hoffmann et al.

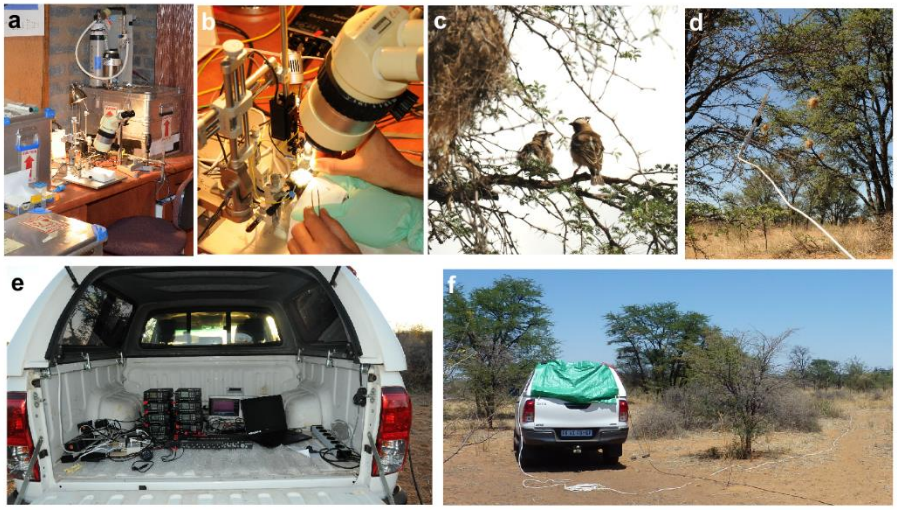

Supplementary Figure 1. Portable electrophysiology laboratory in the Kalahari.
(**a**) depicts the setup used for surgical implantation of electrodes in HVC. (**b**) shows an anesthetized bird fixed inside the stereotaxic device. In (**c**) a pair of *P. mahali* is shown, perching in a tree below their nest. The male bird on the right was equipped with a vocal transmitter on his back and with a neuronal transmitter on its head. (**d**) depicts the antenna used for signal reception, which was placed below a nesting tree. (**e**) shows the setup used for recording of individual vocal and neural signals, which was placed in the trunk of a car that was parked in ~30 m distance to the nesting tree (**f**).

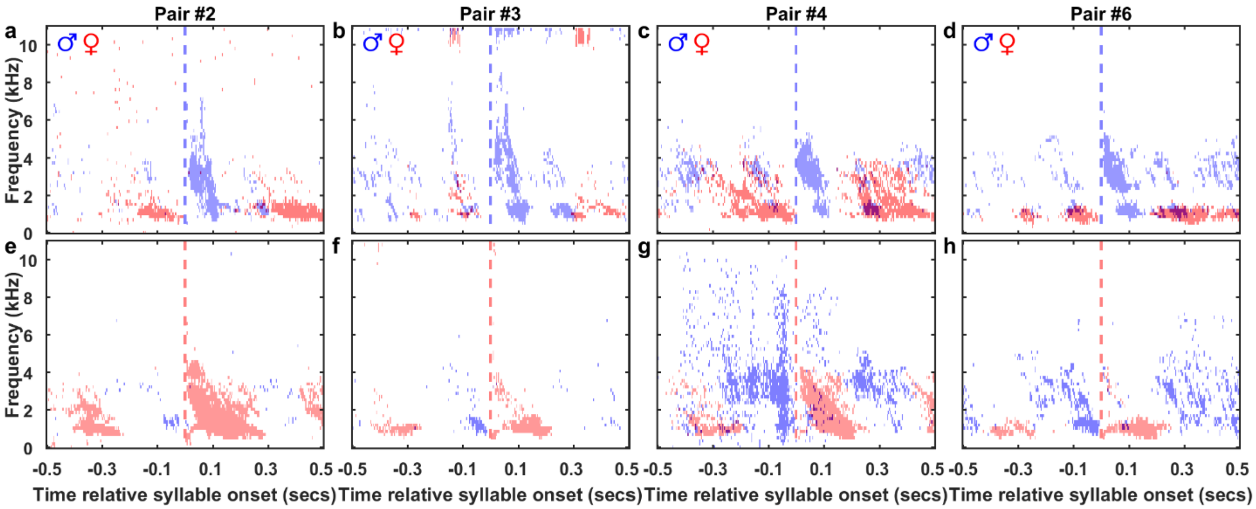

Supplementary Figure 2. Syllable-triggered averaged spectrograms.
Significant (t-test, $p < 0.01$) activity in the averaged spectrogram (see Methods) of male (blue) and female (red) vocal signals within a window between 500 ms before to 500 ms after the onset of male (**a** - **d**) and female (**e** - **h**) duet syllables in duet bouts of three bird pairs is shown by clusters of blue and red time-frequency pixels. The dashed blue and red lines mark the onsets of male and female syllables, respectively, used for generation of the averaged spectrogram. Note the alternating pattern of clusters of red and blue pixels, which indicates that the occurrence of male and female syllables alternated within the duet. Averaged spectrograms of Pair #2, Pair #3, Pair #4 and Pair #6 are based on 1436 male and 1194 female syllables from 194 duet bouts, 421 male and 304 female syllables from 71 duet bouts, 893 male and 554 female syllables from 181 duet bouts and 710 male and 288 female syllables from 131 duet bouts, respectively. For the remaining pairs #1, #5, #7, #8 duet activity (32, 33, 38 and 12 duet bouts, respectively) was too low to generate significant frequency-time pixels in the syllable-triggered averaged spectrograms.

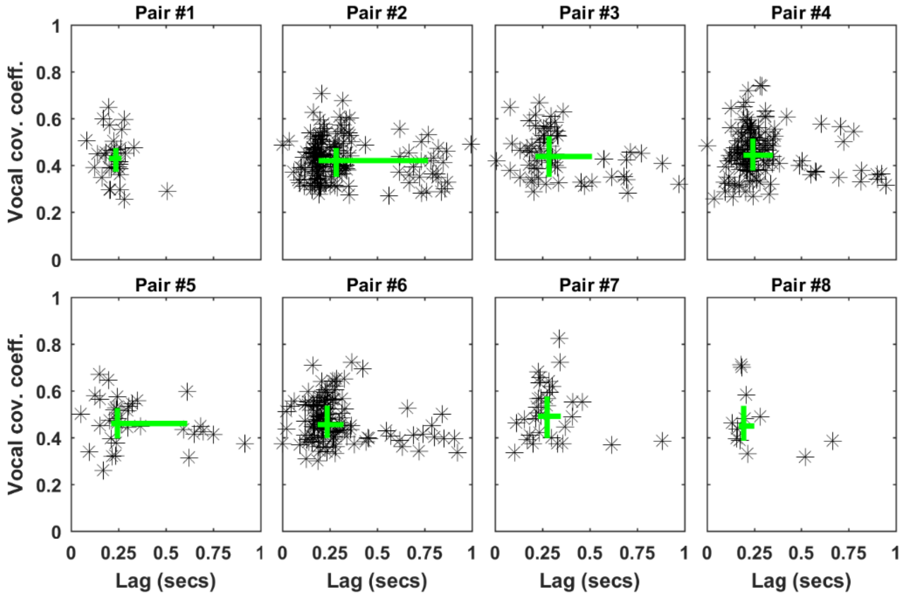

Supplementary Figure 3. Correlation of vocal activity during duetting.
Vocal signals of the male and the female bird of a pair were strongly correlated during duetting at a time shift of ~250 ms. This is indicated by the time lags of maximum cross-covariance between male and female vocal signals (RMS-envelopes) during duetting. The green lines indicate interquartile ranges of lags and cross-covariance coefficients, their intersection is at the medians of the distributions.

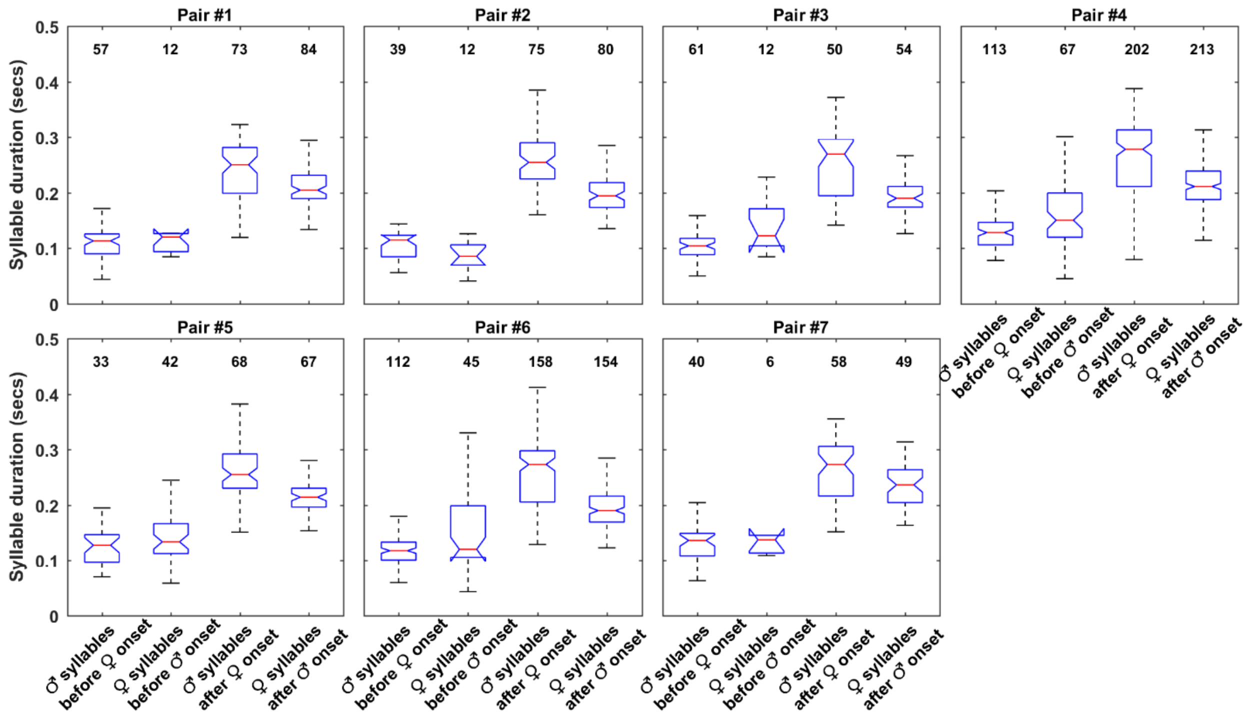

Supplementary Figure 4. Syllable durations.
The duration of syllables sung by a single bird before the second bird's song onset was significantly shorter than the duration of syllables sung in alternation after the second bird's song onset. During duetting, male syllables were significantly longer in duration than female syllables. Boxplots show the distribution of syllable durations for each bird of seven pairs. In the boxplot, the horizontal red line indicates the median, and the bottom and top edges of the box indicate the 25th and 75th percentiles, respectively. The black whiskers extend to the most extreme data points not considered outliers (outliers are not shown). The extremes of the two notches of the box correspond to $y - 1.57(z - x)/\sqrt{n}$ and $y + 1.57(z - x)/\sqrt{n}$, where y is the median, x and z are the 25th and 75th percentiles, respectively, and n is the number of observations. Medians are significantly different at the 5% significance level if the boxes' notches do not overlap. The individual sample size is indicated above each box. The duration of syllables was measured in 15 to 20 duet bouts for each pair of birds. Pair #8 produced only 12 duet bouts during the time of recording and was therefore excluded from this analysis.

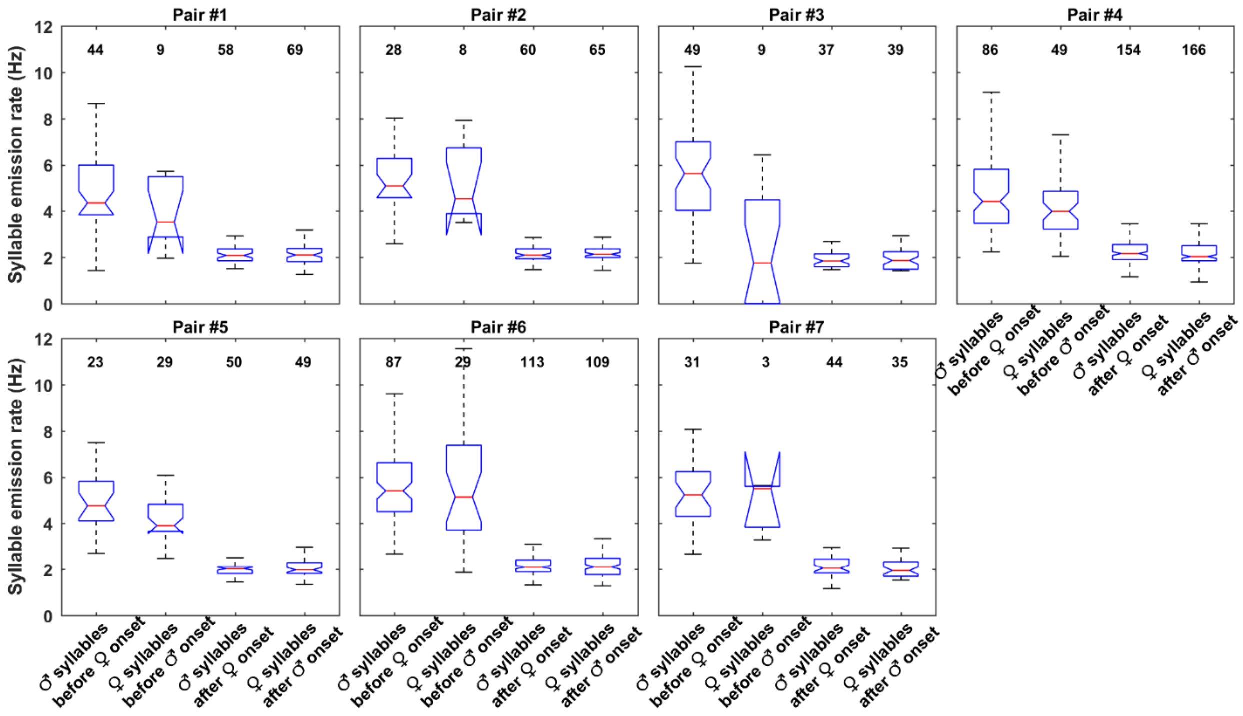

Supplementary Figure 5. Syllable emission rates.

The emission rate of syllables sung by a single bird before the second bird's song onset was significantly higher than the emission rate of syllables sung in alternation after the second bird's song onset. Please see Supplementary Figure 4 for details on box plot labeling. The individual sample size is indicated above each box. The emission rate of syllables was measured in 15 to 20 duet bouts for each pair. Pair #8 produced only 12 duet bouts during the time of recording and was therefore excluded from this analysis.

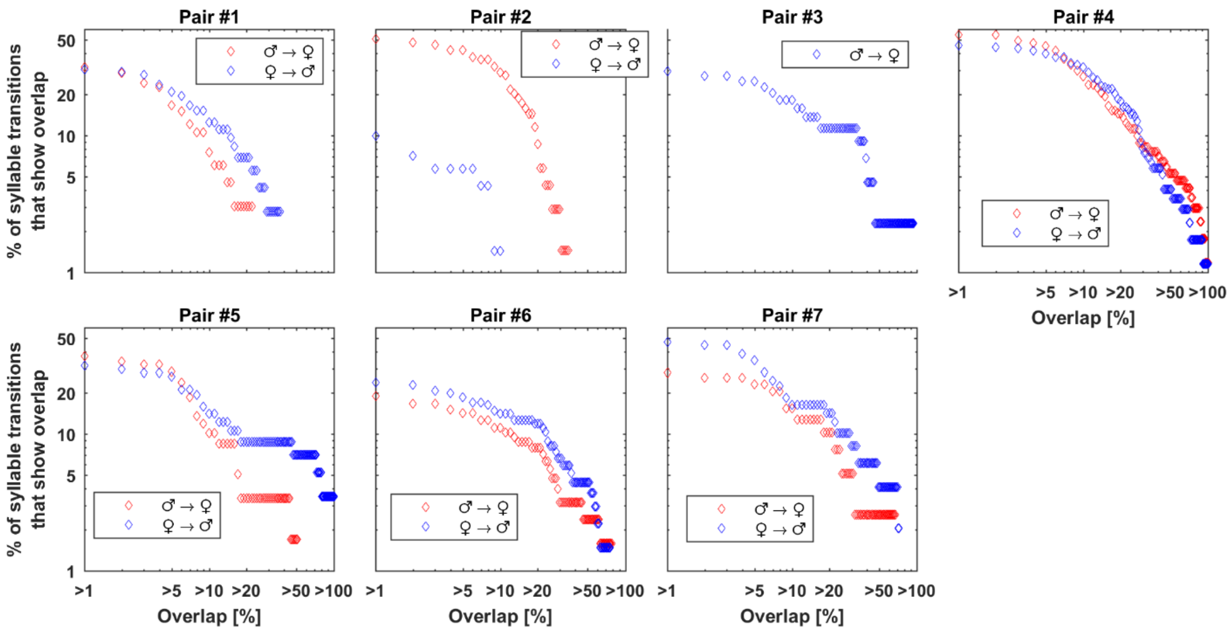

Supplementary Figure 6. Syllable overlaps.

The percentage of duet syllable transitions that showed an overlap larger than the value given on the x-axis is displayed for each bird of seven pairs. Female to male and male to female transitions are marked by blue and red diamonds, respectively. Note that the female bird of Pair #3 never produced syllable overlaps.

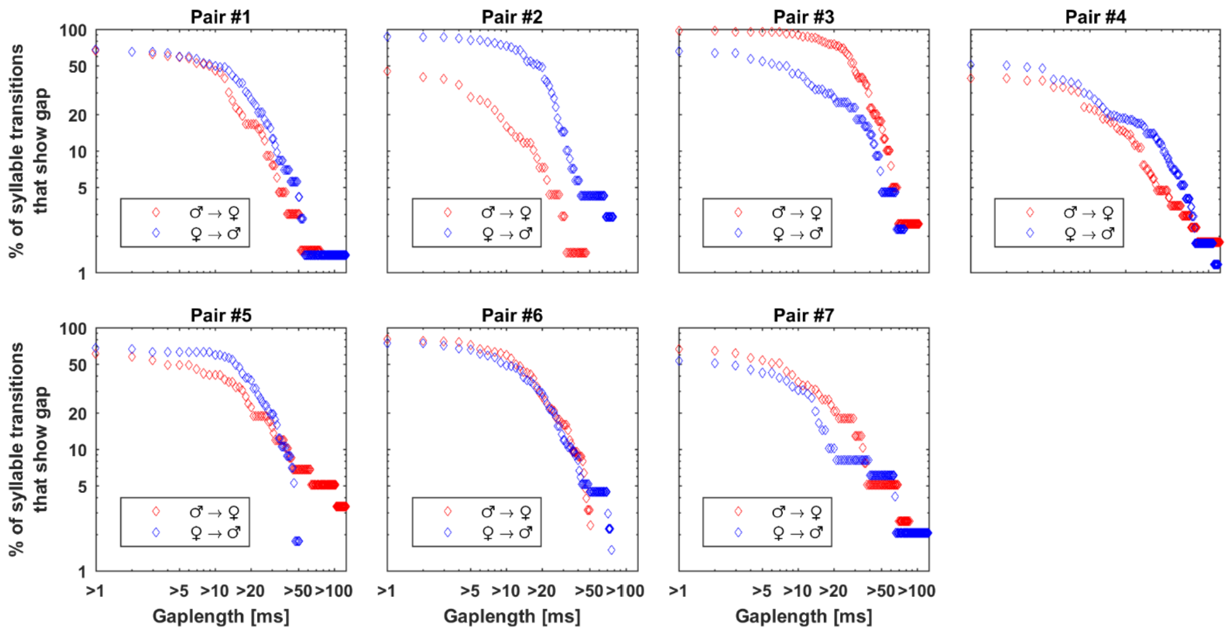

Supplementary Figure 7. Gaps between syllables.
The percentage of duet syllable transitions that showed a gap larger than the value given on the x-axis is displayed for each bird of seven pairs. Female to male and male to female transitions are marked by blue and red diamonds, respectively.

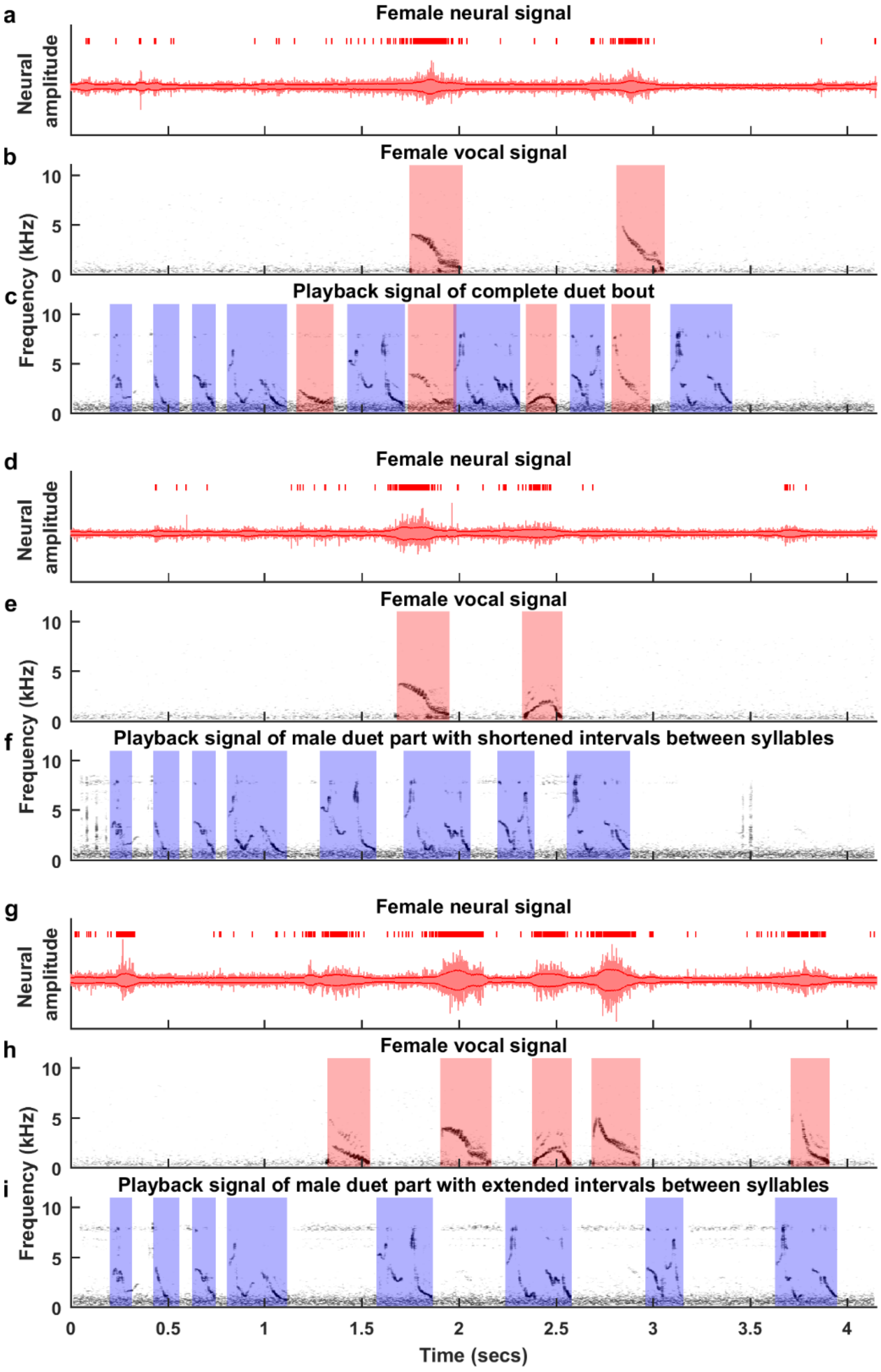

Supplementary Figure 8. Playback experiments.
White-browed sparrow-weavers were able to correctly duet with playbacks of its own duet bouts but was not able to correctly duet with playbacks of own duet bouts with altered temporal patterns. **a**) neural signal and (**b**) spectrogram of vocal signal of a female while duetting with the playback (spectrogram in **c**) of a complete duet bout. **d**) neural signal and (**e**) spectrogram of vocal signal of the same female as it tried to duet with the playback (spectrogram in **f**) of the male duet part with shortened intervals between male duet syllables. **g**) neural signal and (**h**) spectrogram of vocal

signal of the same female as it tried to duet with the playback (spectrogram in **i**) of the male duet part with extended intervals between male duet syllables. Please see the Methods section for details on playback experiments.

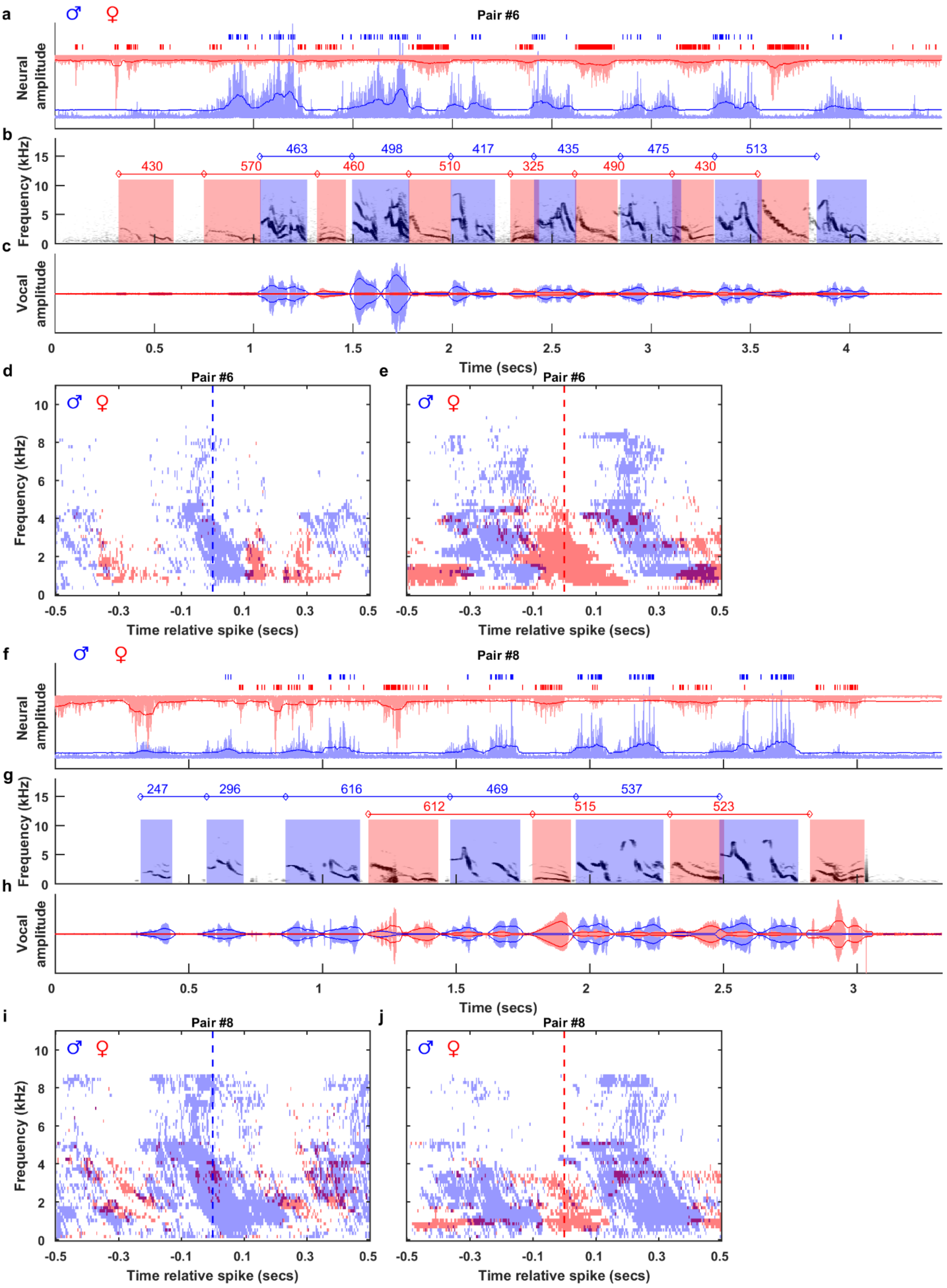
a
♂ ♀
Pair #6
Neural amplitude
b
Frequency (kHz)
463 498 417 435 475 513
430 570 460 510 325 490 430
c
Vocal amplitude
Time (secs)
d
Pair #6
Frequency (kHz)
Time relative spike (secs)
e
Pair #6
Time relative spike (secs)
f
Pair #8
Neural amplitude
g
Frequency (kHz)
247 296 616 469 537
612 515 523
h
Vocal amplitude
Time (secs)
i
Pair #8
Frequency (kHz)
Time relative spike (secs)
j
Pair #8
Time relative spike (secs)

Supplementary Figure 9. Premotor activity in HVC during duetting.

Duet syllables were locked to bursts of premotor neural activity in the HVC of the singing bird, and neural burst alternated between interacting birds. The male and female neural traces (**a** and **f**), and the spectrogram (**b** and **g**) and time signal (**c** and **h**) of male and female vocal traces are shown for duet bouts initiated by the female of pair #6 (**a - c**) or by the male of pair #8 (**f - h**). Male and female syllables and male and female vocal time signals are indicated in blue and red, respectively. Solid dark blue and dark red lines outline the RMS-envelopes (see Methods) of male and female signals, respectively. Spike occurrences in the male and female neural traces are indicated by blue and red short vertical lines, respectively, above the neural signals. Onset-onset intervals male duet syllables and between female duet syllables are given by values above the spectrograms. Significant (t-test, $p < 0.01$) activity in the averaged spectrogram (see Methods) of male (blue) and female (red) vocal signals within a window between 500 ms before to 500 ms after the time of occurrence of male (**d** and **i**) and female (**e** and **j**) spikes in duet bouts of Pair #6 and Pair #8 is shown by clusters of blue and red time-frequency pixels. The dashed blue and red lines mark the time of occurrence of male and female spikes, respectively. Averaged spectrograms of Pair #6 and Pair #8 are based on 420 male and 439 female spikes from 16 duet bouts and 559 male and 411 female spikes from 12 duet bouts, respectively. The spike-triggered averaged spectrogram of Pair #5 is shown in Fig. 4.

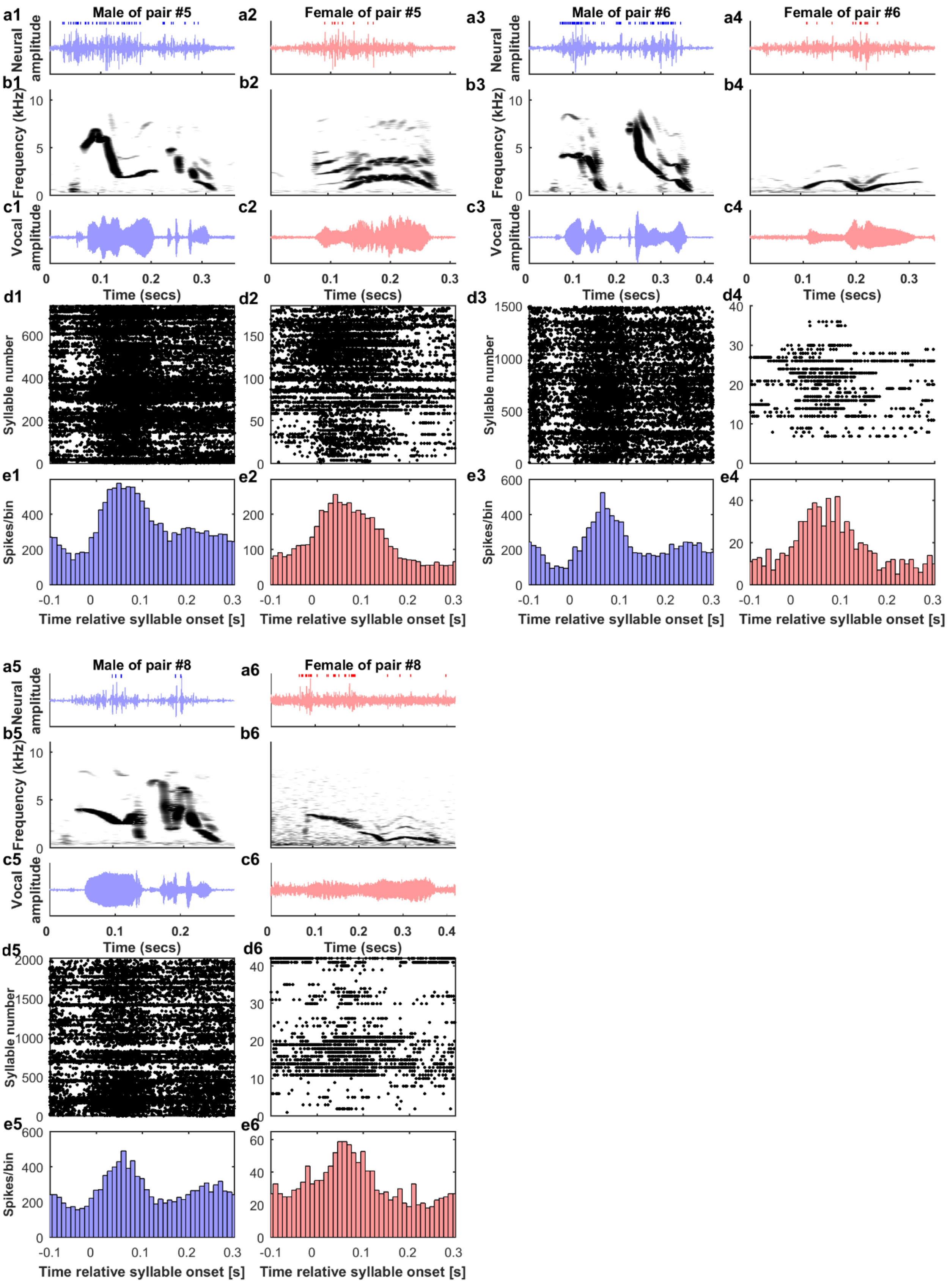
Male of pair #5
Female of pair #5
Male of pair #6
Female of pair #6
Male of pair #8
Female of pair #8
Neural amplitude
Frequency (kHz)
Vocal amplitude
Time (secs)
Syllable number
Spikes/bin
Time relative syllable onset [s]

Supplementary Figure 10. Syllable-triggered representation of neural data.

The filtered neural signal (**a1** – **a6**), spectrogram (**b1** – **b6**) and amplitude waveform (**c1** – **c6**) is shown for one male (blue) and one female (red) exemplary duet syllable for three pairs of *P. mahali*. Spike raster plots (**d1** – **d6**) and corresponding peri-stimulus time histograms (bin size = 0.01 secs, **e1** – **e6**) aligned to the onset of all duet elements within a four-hour recording are shown for the male (blue) and the female (red) bird of three *P. mahali* pairs to demonstrate the correlation between vocalization onset and spike occurrence.

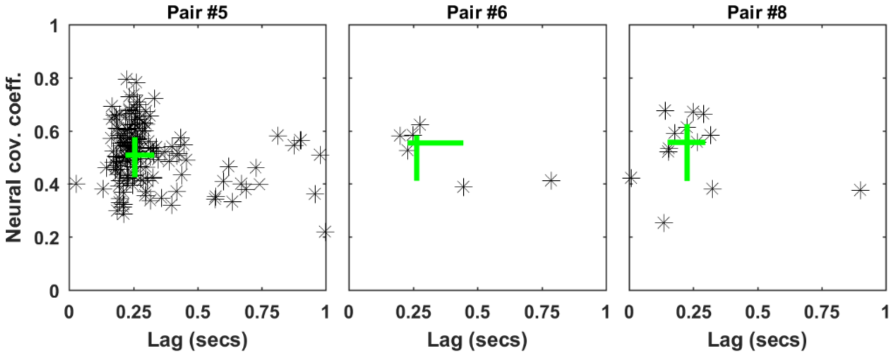

Supplementary Figure 11. Correlation between vocal and neural activity.
Neural signals of the male and the female bird of a pair were strongly correlated during duetting at a time shift of ~250 ms. The black asterisks mark the time lag when the cross-covariance function (see Methods) between the RMS-envelopes of the male and the female neural signal during the alternating part of duet bouts reached its maximum. The interquartile ranges of the covariance coefficients and the time lags are indicated by vertical and horizontal green lines, respectively, and the green lines' intersection marks the distributions' medians.

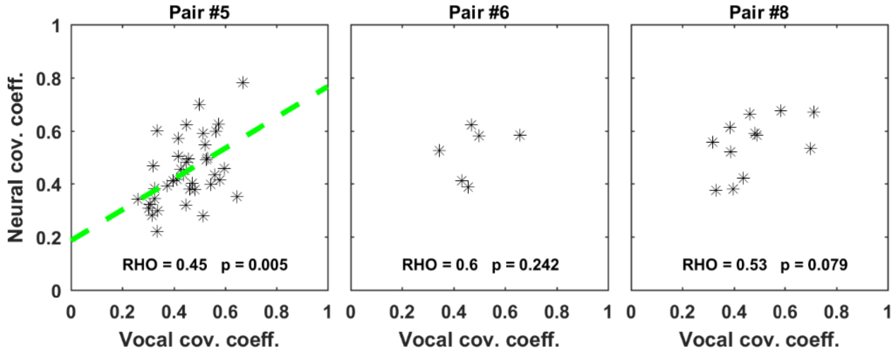

Supplementary Figure 12. The degree of neural synchronization between duet partners determined the degree of duet performance.

Black asterisks mark the coefficient of the cross-covariance function of the RMS-envelopes of male and female neural signals during duetting as a function of the coefficient of the cross-covariance function of the RMS-envelopes of male and female vocal signals during duetting. The green regression line demonstrates the positive correlation of the degree of synchronization between the male and female vocal signals and the degree of synchronization between the male and female neural signals. Spearman's Rho and the significance level of correlation are indicated for each pair.

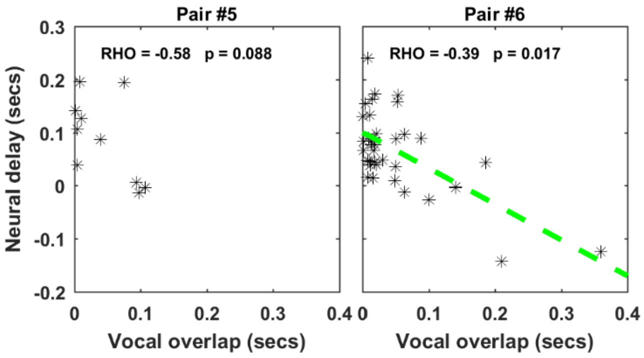

Supplementary Figure 13. The degree of overlap in neural activity was strongly correlated with the degree of syllable overlap.
The dashed green line represents the regression line. n = 10 overlaps produced by Pair #5 and n = 37 overlaps produced by Pair #6.

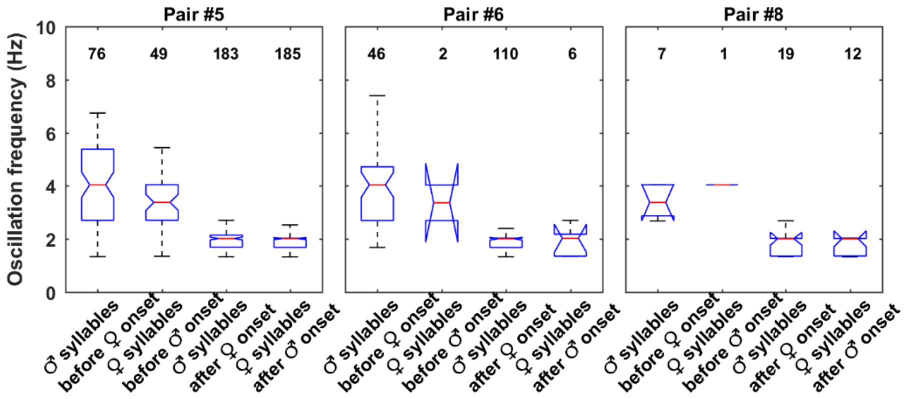

Supplementary Figure 14. Neural oscillation frequencies.

During emission of duet-initiating syllables, oscillation frequencies of male and female neural signals were significantly higher (Mann-Whitney U-test, $p < 0.005$) than during emission of alternating syllables during duetting. Boxplots show the distribution of oscillation frequencies for each bird of three pairs. Please see Supplementary Figure 4 for details on box plot labeling. The number of duet bouts for which the oscillation frequency of neural signals has been determined (see Methods) is indicated above each box.

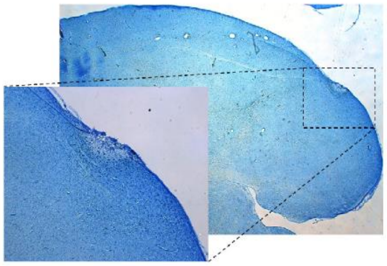

Supplementary Figure 15. Verification of recording sites.
Electrolytic lesions verified the correct positioning of recording electrodes. A Nissl-stained sagittal brain section (see Methods) containing a small lesion in HVC is shown for the male bird of Pair #6.

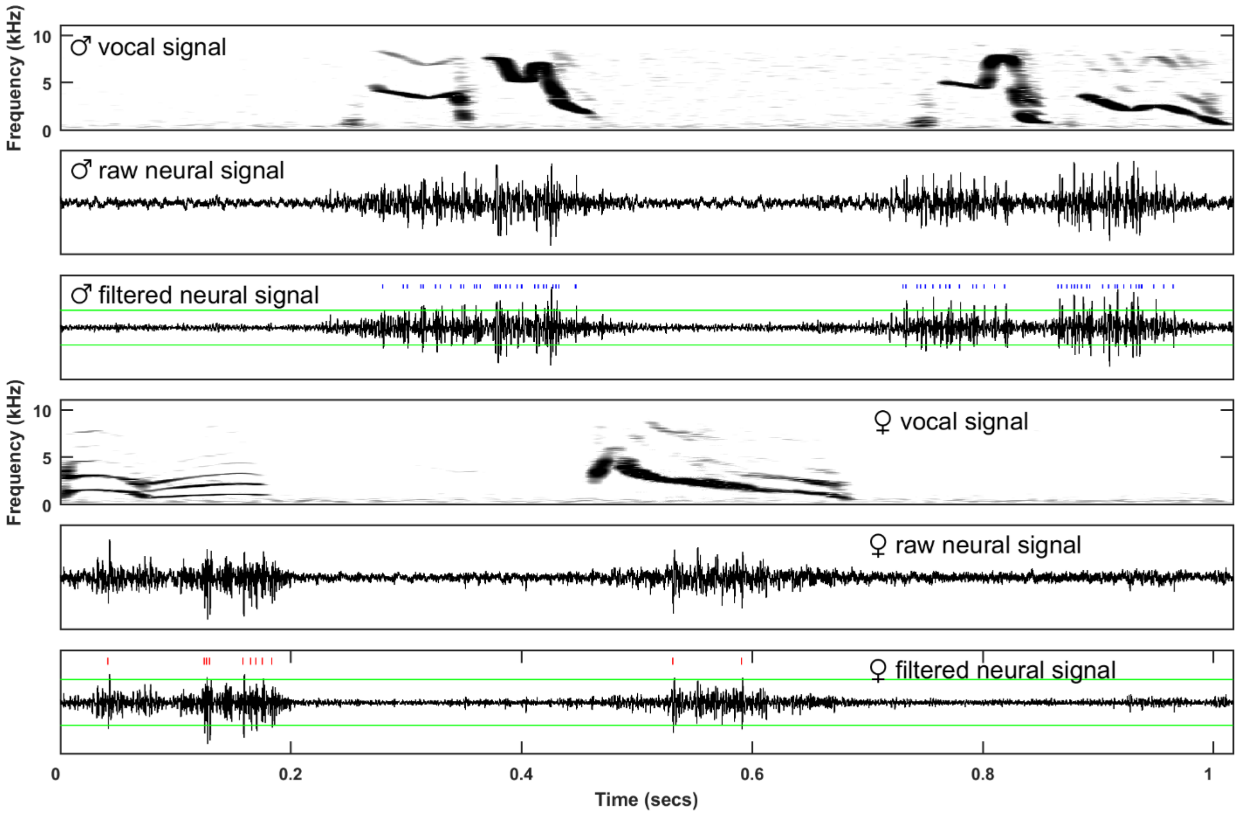

Supplementary Figure 16. Processing steps of neural data.

Cutout of temporally aligned male and female neural and vocal signals recorded from Pair #5 during duetting. From top to bottom: spectrogram of the male vocal signal, amplitude waveform of the raw male neural signal, amplitude waveform of the filtered male neural signal, spectrogram of the female vocal signal, amplitude waveform of the raw female neural signal and amplitude waveform of the filtered female neural signal. In the panels that show waveforms of filtered neural signals, the green horizontal lines indicate voltage thresholds used for spike discrimination, and the small blue and red vertical dashes mark spike occurrences in the male and female filtered neural signal, respectively.

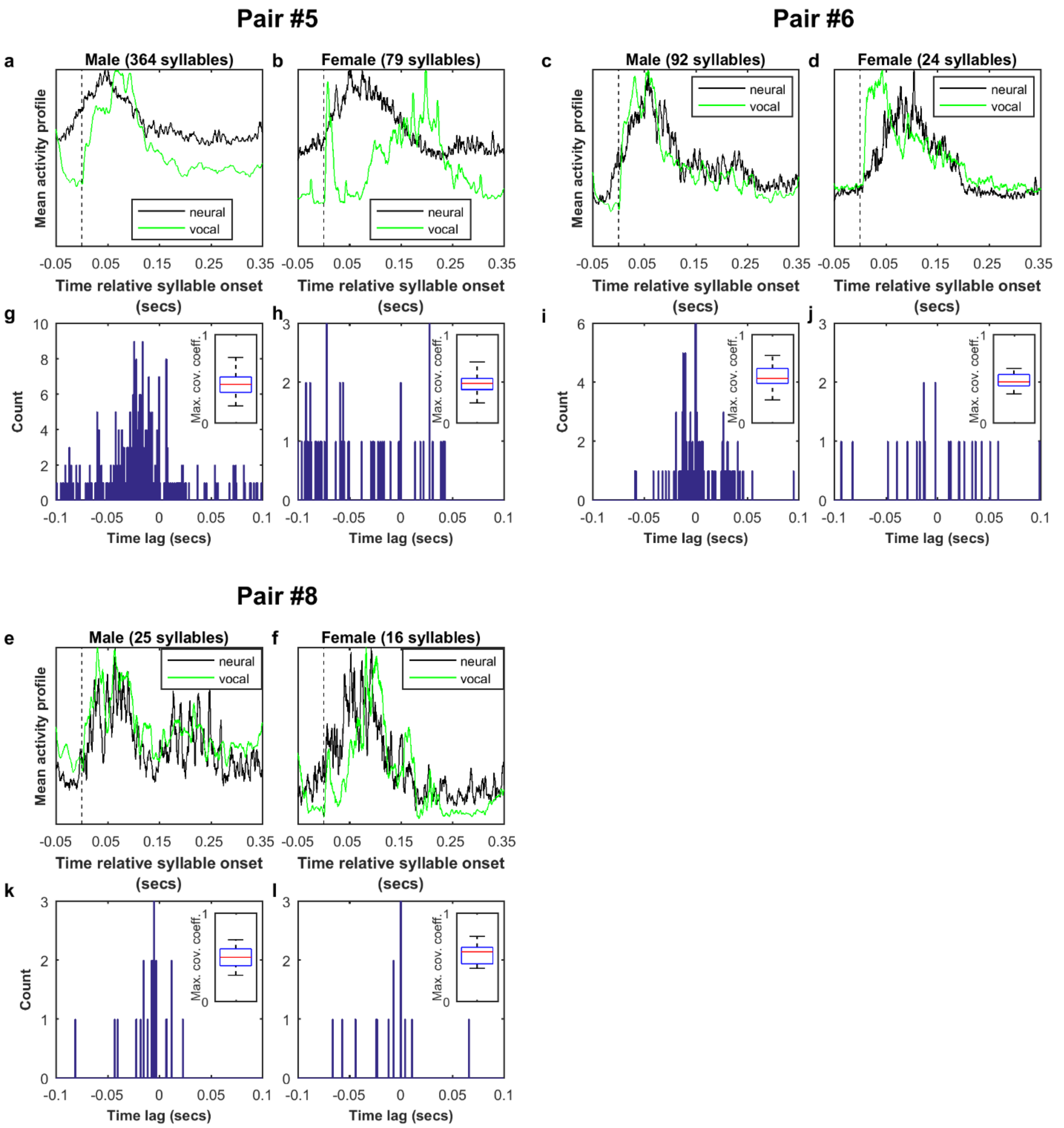

Supplementary Figure 17. Correlation of activity profiles.

For each bird of each pair, the mean activity profile (normalized to maximum values, **a - f**) of filtered neural (black) and vocal (green) signals for the time period between 50 ms before to 350 ms after the onset of all duet syllables that were produced within a 4-hour period of recording. All signals have been rectified and smoothed with a 3-ms sliding window. The dashed vertical lines indicate syllable onsets. It is important to note that the vocal and the neural mean activity profiles were incongruent for each single bird. Histograms in **g - l** show distributions of time lags at which the cross-covariance between the neural and vocal signal during each syllable was maximal. Negative lags = neural signal leading. **Insets** show boxplots of maximum cross-covariance coefficients. Coefficients were normalized to the maximum of the auto-covariance

function. Maximum coefficients were generally low, which indicates that the covariance between the vocal and the neural activity profiles was weak.

	Pair #5		Pair #6		Pair #8	
	male	female	male	female	male	female
**Mean signal to noise ratio (dB)**	5.2	5.1	8.4	6.2	9.6	3.5
**Mean spike rate during singing (spikes s^{-1})**	69.9	84.1	113.3	43.8	25.0	63.0
**Mean spike rate during silence (spikes s^{-1})**	19.8	27.6	35.1	15.8	6.5	5.7

Supplementary Table 1. Signal-to-noise ratios and spike rates of neural recordings.
Mean signal-to-noise ratios and spike rates calculated for duet syllables that were produced within one 4-hour period of recording and for the period of silence of equal duration that followed each syllable.
