# Supplementary material for: Duets recorded in the wild reveal that interindividually coordinated motor control enables cooperative behavior
Source: Nat Commun. 2019 Jun 12;10:2577. doi: 10.1038/s41467-019-10593-3 (PMC6561963; doi:10.1038/s41467-019-10593-3)
Supplement: Supplementary file 2 — Reporting Summary [file 41467_2019_10593_MOESM2_ESM.pdf]

nature research

Corresponding author(s): Susanne Hoffmann

Reporting Summary

Nature Research wishes to improve the reproducibility of the work that we publish. This form provides structure for consistency and transparency in reporting. For further information on Nature Research policies, see Authors & Referees and the Editorial Policy Checklist.

Statistical parameters

When statistical analyses are reported, confirm that the following items are present in the relevant location (e.g. figure legend, table legend, main text, or Methods section).

n/a	Confirmed	
☐	☒	The exact sample size (*n*) for each experimental group/condition, given as a discrete number and unit of measurement
☒	☐	An indication of whether measurements were taken from distinct samples or whether the same sample was measured repeatedly
☐	☒	The statistical test(s) used AND whether they are one- or two-sided<br>*Only common tests should be described solely by name; describe more complex techniques in the Methods section.*
☒	☐	A description of all covariates tested
☒	☐	A description of any assumptions or corrections, such as tests of normality and adjustment for multiple comparisons
☐	☒	A full description of the statistics including central tendency (e.g. means) or other basic estimates (e.g. regression coefficient) AND variation (e.g. standard deviation) or associated estimates of uncertainty (e.g. confidence intervals)
☒	☐	For null hypothesis testing, the test statistic (e.g. *F*, *t*, *r*) with confidence intervals, effect sizes, degrees of freedom and *P* value noted<br>*Give P values as exact values whenever suitable.*
☒	☐	For Bayesian analysis, information on the choice of priors and Markov chain Monte Carlo settings
☒	☐	For hierarchical and complex designs, identification of the appropriate level for tests and full reporting of outcomes
☐	☒	Estimates of effect sizes (e.g. Cohen's *d*, Pearson's *r*), indicating how they were calculated
☒	☐	Clearly defined error bars<br>*State explicitly what error bars represent (e.g. SD, SE, CI)*

Our web collection on statistics for biologists may be useful.

Software and code

Policy information about availability of computer code

Data collection	All digitized signals were recorded in parallel as continuous audio files with a duration of four hours using multichannel software (16-bit, 22050 Hz; ASIO®, Germany).
Data analysis	The software Amadeus® or Audacity® was used to determine the time of duet bout occurrences. Sonograms from vocal signals were assembled from 512 point Fast Fourier Transforms (intel libraries), producing a large number of sonograms each describing syllables or any other supra-threshold sound using custom written (René F. Jansen (Delphi Pascal for Windows) and Andries Ter Maat (CodeWarrior and XCode C++ for Mac OS X)) software. Custom written software (Matlab®, Mathworks, USA) was used for quantitative analysis of behavioral and neural data. The spike sorting software Spike2® (CED, UK) was used for threshold-discriminating spiking activity and subsequent sorting by principal component analysis of their waveform followed by automatic K-means clustering.

For manuscripts utilizing custom algorithms or software that are central to the research but not yet described in published literature, software must be made available to editors/reviewers upon request. We strongly encourage code deposition in a community repository (e.g. GitHub). See the Nature Research guidelines for submitting code & software for further information.

Data

Policy information about availability of data

All manuscripts must include a data availability statement. This statement should provide the following information, where applicable:

- Accession codes, unique identifiers, or web links for publicly available datasets
- A list of figures that have associated raw data
- A description of any restrictions on data availability

The data that support the findings of this study are available from the corresponding author upon reasonable request. Due to the large size of data files, they are not publicly available.

Field-specific reporting

Please select the best fit for your research. If you are not sure, read the appropriate sections before making your selection.

☒ Life sciences ☐ Behavioural & social sciences ☐ Ecological, evolutionary & environmental sciences

For a reference copy of the document with all sections, see nature.com/authors/policies/ReportingSummary-flat.pdf

Life sciences study design

All studies must disclose on these points even when the disclosure is negative.

Sample size	The sample size we chose, was sufficient to enable statistical testing.
Data exclusions	We excluded data for which we only had an incomplete dataset.
Replication	We used conventional methods to produce our data. Therefore they can easily be reproduced.
Randomization	Our study did not include different experimental groups.
Blinding	Blinding was not relevant to our study as our study did not include different experimental groups.

Reporting for specific materials, systems and methods

Materials & experimental systems

n/a	Involved in the study	
☒	☐	Unique biological materials
☒	☐	Antibodies
☒	☐	Eukaryotic cell lines
☒	☐	Palaeontology
☐	☒	Animals and other organisms
☒	☐	Human research participants

Methods

n/a	Involved in the study	
☒	☐	ChIP-seq
☒	☐	Flow cytometry
☒	☐	MRI-based neuroimaging

Animals and other organisms

Policy information about studies involving animals; ARRIVE guidelines recommended for reporting animal research

Laboratory animals	The study did not involve laboratory animals.
Wild animals	We studied 8 male and 8 female adult White-browed sparrow weavers (Plocepasser mahali) near the village of Black Rock, Northern Cape, South Africa (27°7´S, 22°50´E) during February/March 2016 and November/December 2017. All investigated birds were color-banded for individual recognition. Birds were captured shortly after dusk (8-10 pm) inside their roosting nests. The sex was determined by bill color. 5 individuals of each sex were released to their home territory and 3 individuals of each sex were euthanized by an overdose of anesthetics followed by decapitation at the end of the experiments.
Field-collected samples	The study did not involve samples collected from the field.
