# Supplementary material for: Duets recorded in the wild reveal that interindividually coordinated motor control enables cooperative behavior
Source: Nat Commun. 2019 Jun 12;10:2577. doi: 10.1038/s41467-019-10593-3 (PMC6561963; doi:10.1038/s41467-019-10593-3)
Supplement: Supplementary file 3 — Description of Additional Supplementary Files [file 41467_2019_10593_MOESM3_ESM.pdf]

Description of Additional Supplementary Files

File Name: Supplementary Movie 1
Description: In the beginning of the movie, a picture of a male P. mahali foraging below its nesting tree is shown. The bird carries a vocal transmitter on its back (not shown) and a neuronal transmitter on its head. The following short sequence shows a pair of P. mahali performing a duet in their natural environment. Both birds are equipped with vocal and neuronal transmitters. A combination of the vocal signals recorded by both vocal transmitters has been added as sound trace to the movie. The spectrogram of the combined vocal signals (middle panel) and the waveform of the male (top panel) and the female (bottom panel) neural signals are shown in an inset at the bottom of the movie's screen. At the end of the movie, a photograph of a P. mahali flying with a vocal transmitter on its back and a neuronal transmitter on its head is shown. The movie has been edited with the software Shotcut.

File Name: Supplementary Movie 2
Description: The movie shows a pair of free-living P. mahali trying to duet with a playback of a prerecorded duet song, and altered versions of this song (see Methods). Both birds are equipped with vocal and neuronal transmitters. A combination of the birds' vocal signals recorded by vocal transmitters on the birds' backs, and the playback signal recorded by an additional vocal transmitter located close to the speaker that broadcast the playback, has been added as sound trace to the movie. The movie has been edited with the software Shotcut.
